# Supplementary material for: Evaluation of a multicomponent intervention consisting of education and feedback to reduce benzodiazepine prescriptions by general practitioners: The BENZORED hybrid type 1 cluster randomized controlled trial
Source: PLoS Med. 2022 May 6;19(5):e1003983. doi: 10.1371/journal.pmed.1003983 (PMC9075619; doi:10.1371/journal.pmed.1003983)
Supplement: S1 Table — (DOCX) [file pmed.1003983.s004.docx]

**S1 Table. Subgroup analysis of effectiveness of the intervention at 12 month follow-up by health districts.**

|  |  | | **Mean differences (95% CI)** |  | | **Estimated ITT**  **Mean differences (95% CI)** | |  |  |
| --- | --- | --- | --- | --- | --- | --- | --- | --- | --- |
| **Primary Outcome** | | | |  | | | |  |  |
| DDD per 1000 inhabitants per day | | | | | | | | |  |
| Valencia (Conselleria Salut Univ.; Arnau de Vilanova llíria district) | **I:**89.7±32.0  **C:**85,9±34.6 | -3.3  (-7.5;1.2) p=0.155 |  |  | -3.3  (-7.5;1.2)  p=0.156 | |  | | |
| Balearic Islands (IbSalut) | **I:**67.2±28.9  **C:**73.4±30.5 | -2.7  (-4.2;-1.3)  p <0.001 |  |  | -2.8  (-4,3;-1.3)  p <0.001 | |  |  |  |
| Catalonia (Institut Català de la Salut;Tarragona-Reus district) | **I:**71.7±28  **C:**80.3±31.4 | -6.1  (-11.4;-0.9)  p=0.022 |  |  | -6.8  (-12,2;-1,30)  p =0.023 | |  |  |  |
| Total | **I:**71.0±29.9  **C:**76.5±31.5 | -3.4  (-5.1;-1.8) |  |  | -3.3  (-4.87;-1.65) | |  |  |  |
| **Secondary outcome** | | | |  | | | |  |  |
| Proportion of long-term BZD users (>6 months) | | | | | | | | |  |
| Valencia (Conselleria Salut Univ.; Arnau de Vilanova llíria district) | **I:**14.7±3.2  **C:**14.7±3.3 | -0.62  (-1.18;-0.06)  p=0.030 |  |  | -0.62  (-1.18;-0.06)  p=0.029 | |  | | |
| Balearic Islands (IbSalut) | **I:**8.2±3.0  **C:**8.9±3.5 | -0.33  (-0.52;-0.14)  p=0.001 |  |  | -0,32  (-0,51;-0,13)  p<0.001 | |  |  |  |
| Catalonia (Institut Català de la Salut;Tarragona-Reus district) | **I:**8.4±2.4  **C:**9.5±2.7 | -0.52  (-1.17;0.14)  p=0.123 |  |  | -0.49  (-1.13;0.14)  p=0.124 | |  |  |  |
| Total | **I:**9.1±3.6  **C:**9. 8±3.8 | -0.38  (-0.57;-0.18) |  |  | -0.39  (-0.58;-0.19) | |  |  |  |
| Proportion of long-term BZD users (>6 months) in patients >65 yrs | | | | | | | | |  |
| Valencia (Conselleria Salut Univl.; Arnau de Vilanova llíria district) | **I:**32.7±6.7  **C:**32.5±7.1 | -0.70  (-2.70;1.29)  p=0.490 |  |  | -0.70  (-2.70;1.29)  p =0.489 | |  | | |
| Balearic Islands (IbSalut) | **I:**23.5±7.1  **C:**25.1±7.6 | -0.93  (-1.39;-0.48)  p <0.001 |  |  | -0.92  (-1.38;-0.47)  p <0.001 | |  |  |  |
| Catalonia (Institut Català de la Salut;Tarragona-Reus district) | **I:**20.2±5.8  **C:**21.2±6.1 | -0.82  (-2.44; 0.80)  p=0.332 |  |  | -0.68  (-2.30; 0.93)  P=0.324 | |  |  |  |
| Total | **I:**23.9±7.7  **C:**25.2±7.9 | -0.90  (-1.46;-0.34) |  |  | -0.87  (-1.35;-0.26) | |  |  |  |

Abbreviations: I= Intervention; C=Control
