# Supplementary material for: Evaluation of a multicomponent intervention consisting of education and feedback to reduce benzodiazepine prescriptions by general practitioners: The BENZORED hybrid type 1 cluster randomized controlled trial
Source: PLoS Med. 2022 May 6;19(5):e1003983. doi: 10.1371/journal.pmed.1003983 (PMC9075619; doi:10.1371/journal.pmed.1003983)
Supplement: S1 Study Protocol — (DOC) [file pmed.1003983.s005.doc]

**STUDY PROTOCOL**

**Evaluation of a multifactorial intervention to reduce the benzodiazepine consumption in primary care. Cluster randomized clinical trial.**

**Código: PI15/01480**

Gerencia de Atención Primaria

C/ Reina Esclaramunda

07002 Palma de Mallorca

Teléfono 971 17 58 92.

**Principal Investigator: Catalina Vicens Caldentey**

**ATC Therapeutic Groups included in the study:**

N05BA (diazepam, chlordiazepoxide, potassium clorazepate, lorazepam, bromazepam, clobazam, ketazolam, alprazolam, halazepam, pinazepam, clotiazepam, bentazepam),

N05CD (flurazepam, flunitrazepam, triazolam, lormetazepam, midazolam, brotizolam, quazepam, lorprazolam) and N05CF (zopiclone, zolpidem, zaleplon) groups of the Anatomical Therapeutic Chemical (ATC)

**Evaluation of a multifactorial intervention to reduce the benzodiazepine consumption in primary care. Cluster randomized clinical trial.**

**ABSTRACT:**

**Objective**: To evaluate the efficacy of a multifactorial intervention targeted to General Practitioners (GPs) on adequacy of benzodiazepine (BZD) prescription and approach to discontinue their use in long-term consumers.

Design: Randomized clinical trial stratified by clusters where randomization units will be the health centers (PHC).

**Sample size**: The participation of 508 GPS will be necessary to demonstrate a reduction of at least 10% in doses per 1,000 inhabitants per day (DHD) compared to the control group.

**Study subjects**: GPS from primary health care centers (PHC) in the Balearic Islands, in the Arnau de Vilanova-Liria sector (Valencia) and in Tarragona-Reus who have accepted by consensus of the majority of the team to participate in the study. Participants GPs will be randomized to the intervention or control arm.

**Intervention**: A multcomponent intervention targeted to GPs on the adequacy of the benzodiazepine prescription (indication, dosage, duration) and an educational intervention based on tapering off long-term BZD use, a monthly BZD prescription feedback and access to a support webpage.

**Measurements:** DHD of benzodiazepines by GP at 12 months, evolution and comparison with the DHD by participating health centers. Percentage of total patients and patients over 65 years of age with long-term use of benzodiazepines (≥ 6 months).

The DHDs will be obtained from the health service's pharmacy billing system and chronic consumers from the electronic prescription database. Feasibility, acceptability, adoption and fidelity of the intervention through a questionnaire to the GPs of the intervention group.

**INTRODUCTION**

Benzodiazepines are a group of drugs acting on the central nervous system through the gamma-aminobutyric acid (GABA) inhibitory system. They have hypnotic, anxiolytic, muscle relaxant and anticonvulsant properties (1), the pharmacological predominating action depends on the pharmacokinetic characteristics of each drug.

They are effective in the short-term however their long-term efficacy is still unclear. Long-term use is not recommended due to their ability to produce tolerance and dependence. Physical and psychological dependence may occur after a few weeks of starting the treatment, with increasing risk with higher doses and longer is the treatment. When BZD are abruptly stopped, even at therapeutic doses, it can trigger withdrawal symptoms characterized by insomnia, irritability, anxiety, and even seizures (2). Benzodiazepine analogues (zolpidem, zopiclone and zaleplon) are indicated for the short-term treatment of insomnia and although they were developed to improve some adverse effects of benzodiazepines, there is no clear evidence that they behave differently from short half-life benzodiazepines (3).

Morover BZD use is associated with many adverse effects, including somnolence, daytime drowsiness, memory disruption, increased risk of falls resulting in hip fracture and motor vehicle accidents (5,6,7). Other studies expressed concerns about possible links of long-term BZD use with mortality (8,9,10). Long-term BZD use is considered inappropriate for people aged 65 years or more by Beers Criteria of the American Geriatric Society (11) and recommend avoiding their use for insomnia.

Health authorities and therapeutic guidelines recommend the use of benzodiazepines only for short periods of time. The Therapeutic Prescription guide adapted from the British National Formulary edited by the Ministry of Health and Consumption, stated that “benzodiazepines are indicated for the relief, for a short period (2-4 weeks), of anxiety or an intense and invalidating insomnia when it makes the patient suffer exaggeratedly…”, the use of benzodiazepines for the treatment of “mild and anxiety is inappropriate and inadequate” and on the other hand the Clinical Practice Guideline for the Management of Patients with Anxiety Disorders (12) in Primary Care and the Clinical Practice Guideline for the management of patients with insomnia (13) always recommend using the minimum effective dose, in monotherapy and with a duration that does not exceed 2-4 weeks, both consider it important to restrict their use to acute situations and highlight the lack of efficacy of benzodiazepines in these indications when used in the long-term.

Despite these recommendations benzodiazepines are one of the most consumed pharmacological groups in Spain, as in most Western countries. However, in many European countries, such as France, Denmark, Holland, Sweden... in recent years, consumption has been stabilizing and even decreasing, while in our country, as in Portugal, consumption continues to increase(14).

The DDD is the average daily dose when a drug is used in its main indication. The number of DDDs consumed in a geographical area is expressed per 1,000 inhabitants and per day and is the Daily Inhabitant Dose (DHD). This parameter can provide an estimate of the number of patients treated daily with a given drug and is very useful for conducting comparative studies between prescribers or areas, since it avoids the variability of other forms of drug consumption measurement, such as the number of containers. For this reason, we have decided to use this unit to measure the evolution of consumption over time and compare between professionals and health centers.

According to data from the AEMPS Medication Use Observatory (15), in 2012 about 90 DHDs of benzodiazepines were consumed in Spain by the Public Health System. This means that for every 1,000 inhabitants, a hypothetical 90 consumed a daily dose of a benzodiazepine every day of the year. Although not exactly, this data brings us closer to the prevalence of its consumption, which in this case would be around 9% of the Spanish population. In addition, between 2000 and 2012, the global consumption of anxiolytics and hypnotics increased from 56.7 to 89.3 DHD, this represents an increase of 57.4%, which was greater for hypnotics (N05C) than for anxiolytics (N05B).

The latest National Health Survey carried out by the Ministry of Health in 2012 reveals that 17% of those surveyed had been taking tranquilizers or hypnotics in the two weeks prior to the interview, with significant variability according to sex and age. The proportion of women who consume benzodiazepines is double than that of men and increases considerably with age. In the group of women over 65 years, this percentage rises to 36.6%(16).

Nearly 3 out of every four prescriptions for benzodiazepines are initiated by the GPs in the primary care setting (17), where the follow-up and control of the treatment is usually carried out. Primary care physicians daily face a heavy workload with little time per consultation and a growing demand for care due to stressful life processes that generate suffering and emotional discomfort in their patients. The need to give rapid relief to the patient's symptoms favours medicalization. It is common for any of these demands to indicate treatment with a benzodiazepine and, due to several factors depending on the patient, the professional or the organization, the medication is maintained long enough to generate pharmacological dependence. Once dependence has been established, the appearance of withdrawal symptoms when treatment is discontinued encourages treatment to be prolonged over time, and often this is the only reason to maintain BZD consumption.

In the international scientific literature, there is consensus that the best way to withdraw benzodiazepines is gradually and in stages, in several countries such as the United Kingdom, Holland, Canada and Spain, several studies have been carried out trying to evaluate the effectiveness of different interventions or strategies designed to reduce the chronic consumption of benzodiazepines, some of them have been evaluated in clinical trials and several meta-analyses have compared them with each other (19,20,21).

Patients may be reluctant to cease BZD treatment due to anticipation of withdrawal symptoms and often GPs, although aware about the risks of long-term use, may feel a lack of confidence in managing benzodiazepine withdrawal and are worried about possible patient discomfort or relapse of their symptoms when discontinuing treatment. These doubts on both sides, patients and professionals favour the perpetuation of the treatment (18).

In the international scientific literature, there is consensus that the best way to withdraw benzodiazepines is gradually and in stages, in several countries such as the United Kingdom, Holland, Canada and Spain. There is evidence of the effectiveness of interventions or strategies designed to reduce the chronic consumption of benzodiazepines (19,20,21).

The research team of this project designed the recently published BENZORED (22) project carried out by GPs from three Spanish regions (Balearic Islands, Catalonia and Valencian Community) with the aim of evaluating the efficacy of two interventions in primary care to reduce long-term use of benzodiazepines. The study was funded with a grant from the Health Research Fund of the Carlos III Health Institute (PS09/00947).

In this study, two interventions were evaluated consisted of a structured interview to discuss with patients the benefits and risks of prolonged BZD use, followed by gradual taper of the medication. One of the two interventions was accompanied by follow-up visits every 2-3 weeks and, in the second, written information was given to the patient with the medication withdrawal schedule and without follow-up visits being scheduled. The two interventions were compared with usual care and achieved 45% of patients stopped the BZD consumption compared with 15% of the usual care group. The safety of the interventions was also evaluated by assessing the levels of anxiety, depression, dissatisfaction with the quality of sleep or alcohol consumption The conclusions of the study were that both interventions were three times more effective than the usual care to discontinue benzodiazepine use and that patients did not increase the levels of anxiety, depression, dissatisfaction with sleep or alcohol consumption. Since the two interventions have similar efficacy and given that carrying out several follow-up visits increases the complexity of the intervention, the most efficient strategy could be to carry out the intervention with a written instructions with gradual taper schedule and without follow-up visits.

This study has led us to have a tool recognized as effective and feasible to reduce the long-term use of benzodiazepines, and consequently our intention is to go one step further with this project, which consists of evaluating the implementation of this strategy in a wide health area to assess its impact in the reduction of benzodiazepine use.

The objective of this study is, therefore, to evaluate the impact of implementing a multifactorial, training and feedback intervention based on the strategy used in the previous study, targeted to GPs from three Spanish regions to reduce benzodiazepine prescriptions.

**HYPOTHESIS AND OBJECTIVES**

**HYPOTHESIS**

We consider the following conceptual hypothesis: a multifactorial intervention that includes a training strategy for GPs on appropriateness in the prescription and scheduled cessation of benzodiazepines for patients with chronic consumption and a monthly graphical information about benzodiazepine prescription (audit and feed-back)t is effective in reducing the total prescription of benzodiazepines by GPs and reduces the number of chronic users.

The proposed multifactorial intervention reduces the prescription of benzodiazepines by at least 10%, measured in DHDs, in the intervention group compared to the control group at 12 months.

The second operative hypothesis is that the intervention reduce the number of patients with chronic consumption of benzodiazepines (defined as daily consumption during the last 6 months) and especially in people over 65 years of age.

Our last hypothesis affirms that the proposed intervention is feasible, acceptable, easily adoptable and the fidelity of the doctors to the intervention is optimal.

**OBJECTIVES**

To evaluate the efficacy of a multifactorial intervention targeted to GPs to.

1. Reduce total prescription of benzodiazepines measured in DHD 12 months after the intervention compared to the control group.
2. Reduce the prevalence of patients with BZD long-term use as well as the prevalence long-term users aged 65 years or more at 12 months after the intervention.
3. A final objective will be to analyze the feasibility, acceptability, adoption and fidelity of the intervention.

**METHODS**

**STUDY DESIGN**

Randomized clinical trial stratified by clusters where randomization units will be all PHC from Balearic Islands, the Arnau de Vilanova-Liria district (Valencian Community) and Tarragona-Reus district (Catalonia). A cluster design will be used to avoid a possible contamination bias between doctors. Randomization will be stratified by baseline DHD consumption, health sector and proportion of population aged 65 or more.

**RANDOMISATION**

Once the PHC accept participation, PHCs will be randomized to a control or intervention group through a randomization process stratified by mean baseline BZD consumption in DHD, population over 65 years of age and by health district

In order to avoid contamination bias, the randomization unit will be the health center and to guarantee the comparability of the control and intervention groups, it will be carried out in a stratified way. Randomization will be performed in blocks of 4 by generating a sequence of random numbers (Epidat 3.1 program) for each of the two arms of the trial.

In the intervention PHCs the training will begin and they will receive all the components of the intervention and in Control PHCs no changes in their usual care practice.

**SETTING. STUDY POPULATION**

The study will be developed in all PHC belonging to Balearic Islands, the Arnau de Vilanova-Liria district (Valencian Comunity) and the Tarragona-Reus district in Catalonia.

Study population: GPs belonging to PHCs from Mallorca, Menorca and Ibiza/Formentera, the Arnau de Vilanova-Liria district (Valencian Community) and the Tarragona-Reus district (Catalonia) who, by a majority of 2/3 of the team agree to participate in the study.

- Inclusion criteria: Physicians belonging to health centers in the study area who agree to participate.

- Exclusion criteria: No exclusion criteria

**SAMPLE SIZE**

The published data of the AEMPS drug observatory mean DHD of BZD consumption of 89.3, has been used. The billing data of the Balearic Islands have been used to calculate the standard deviation and the intraclass correlation coefficient of this parameter. For an alpha risk of 5% and a beta risk of 20% and a reduction of at least 10% of the predicted mean DHD, it is necessary that 350 primary care physicians participate (175 in each arm of the trial).

To correct this number for the type of design used (cluster randomization) we used the formula (Ukoumunne OC;Health Technol Assess. 1999;3(5):iii-92): Deff = 1 + (m - 1) * ICC. Where Deff corresponds to the design effect, m to the size of the clusters and ICC to the intraclass correlation coefficient. The ICC has been calculated from the billing data for the Balearic Islands and is ICC=0.05, on average a health center is made up of 10 PC doctors, m=10, the Deff will therefore be 1.45 . Thus, the necessary subjects in each group will be 1.45*175=254, the total sample size is 508.

A part of the budget requested in this project will be used for quality control and monitoring visits. Before the start of the study, the protocol, questionnaires, the project investigator's booklet, the procedures to obtain informed consent and the procedures for the recruitment, the e-mailing the feed-back information to GPs, monitoring the intervention, and verification of the data extracted from the electronic prescription database systems of each Region. Each Region will ensure compliance with data protection regulations.

**IntervenTION**

The intervention will be applied in PHC that have agreed to participate and signed the team's commitment. The intervention consists of a 2-hour initiating a benzodiazepine prescription focusing on adjusting the dose and setting the duration of the treatment when starting the prescription. The approach to discontinue benzodiazepine use for long-term users who are candidates to be withdrawn. The workshop consists of a theoretical-practical training given by members of the research team with experience training in this field.

The second component of the intervention consists of audit and feed-back: monthly graphical information about the own benzodiazepine’s prescription, the prescription of the PHC and the global BZD prescription of the Region in DHD will be e-mailed to each GP of the intervention Group.

In addition to face-to-face training, and the audit and feed-back, a web-based page will be created for GPS with supporting documentation and information on benzodiazepine prescription and withdrawal in an easy-to-read format with access to examples and clinical cases based on real cases. GPs in the intervention group will have access to this information at any time during the study.

**DEVELOPMENT OF THE STUDY.**

In a first phase, the acceptance of the PHC Heads to participate in the study will be collected, in these centers all the GPs will be invited to participate, and once the agreement of a majority of 2/3 of the team is obtained, they will sign the participation commitment. If any GP explicitly informs that he does not want to participate in the project, not receive information about his prescription, he will be excluded from the study. At the same time, the percentage of patients aged 65 years or older will be collected as well as a baseline determination of the prescription of benzodiazepines in each center in DHD. PHC will be randomized according to these baseline characteristics.

Pilot study: The study will begin in two control PHC and two intervention PHC of each Region to assess the acceptability and initial feasibility of the intervention, the logistics of the study, as well as the viability and sustainability of the intervention and adherence. Possible aspects to be improved will be collected before starting the study in PHCs.

In addition, four semi-structured interviews will be conducted with the professionals who participate in this phase of the study to obtain their opinion on the intervention carried out.

Analysis of the effectiveness of the intervention and final results of the implementation. Once the intervention has been adapted to the acceptability and feasibility criteria established in the pilot study and the information systems have been adapted, the logistics have been fine-tuned, the study will start in the rest of the PHCs.

The inclusion of PHCs, randomization and the workshop training will be carried out over a period of 6-8 months in a staggered manner at a rate of 6-8 PHCs each month. The intervention centers will receive training as they are included in the study. The control group will not receive training or specific instructions, they will be informed of their condition as control centers and information will be collected on sociodemographic and general characteristics of the PHCs and the GPs.

The information about the BZD prescription will be e-mailed in DHD to each GP of the intervention group monthly during the 12 months following the intervention. The information on the number of chronic consumers, both total and over 65 years of age, will be collected at baseline prior to the intervention and after 12 months.

The acceptability, feasibility, adoption and fidelity questionnaires will be carried out at 12 months only to the professionals of the intervention group.

**PRIMARY OUTCOME**

Prescription of benzodiazepines by GP and PHC at 12 months after the intervention measured in Doses per 1000 inhabitants per year (DHD).

The identification of the different pharmaceutical specialties will be made from a list of all the presentations of the drugs of the groups N05BA (anxiolytics), N05CD and N05CF (hypnotics and sedatives) of the ATC classification (Anatomical, Therapeutic, Chemical Classification System) of the WHO.

Source: Electronical prescription database of each Spanish Region participating in the study.

**SECONDARY OUTCOMES**

Percentage of patients with long-term BZD use during the last 6 months measured at 12 months from the intervention. Long-term is defined as taking BZD for at least 6 months.

Percentage of patients aged 65 years or older with long-term BZD use during the last 6 months measured at 12 months from the intervention. Long-term is defined as taking BZD for at least 6 months.

Feasibility, defined as the extent to which an intervention is carried out in a given context or organization, will be assessed using the intervention facilitator subscale of the SAFE questionnaire (Bird VJ, Le Boutillier C, Leamy M, Williams J, Bradstreet S, Slade M (2014) Evaluating the feasibility of complex interventions in mental health services: standardized measure and reporting guidelines, British Journal of Psychiatry) adapted to the present study. GP questionnaire to the intervention group.

Acceptability: defined as the perception among the actors/stakeholders that an intervention is acceptable/convenient. The Devilly GJ, Borkovec TD questionnaire will be used. Psychometric properties of the credibility/expectancy questionnaire. J Behav Ther Exp Psychiatry. 2000 Jun;31(2):73-86.) adapted to the present study, and a questionnaire designed ad hoc on a Likert scale of 5categories on the degree of agreement with the different components of the intervention and the intervention as a whole. GP questionnaire to the intervention group.

Adoption: defined as the intention, initial decision, or action of trying to use a new intervention. The percentage of physicians in the intervention group who answered affirmatively to the question about whether they had used the proposed intervention will be collected. They will also be asked about the degree of agreement with the possibility of continuing to use the intervention in the future and if they would recommend carrying out this intervention. intervention to their peers, for which an ad hoc questionnaire designed on a 5-category Likert scale will be used. GP questionnaire to the intervention group.

Fidelity: defined as the degree to which an intervention was implemented as it had been designed, they will be invited to respond on the approximate percentage of patients with chronic consumption to whom weaning has been proposed and the percentage of patients to whom it has been proposed. has reported on the duration of treatment in the prescription of benzodiazepines. GP questionnaire to the intervention group.

INDEPENDENT VARIABLES:

Arm: Control group or Intervention group.

Socio-demographic variables of the professionals: age, sex, specialty in family and community medicine and years of experience will be collected. Information source: GP questionnaire.

Baseline characteristics of the number of patients: total patients, percentage of women, and those over 65 years of age. Source: Primary care information systems.

STATISTYCAL ANALYSIS

All data will be analyzed on an intention-to-treat basis, which means that all physicians regardless of whether or not they attended the training sessions will be analyzed.

The prescription data and the number of chronic users will be collected at the GP level. No data will be available that allows individual identification of patients and GPs will keep their encryption code for statistical analysis. For the statistical analysis, the SPSS for Windows v.15 program will be used.

Descriptive analysis, labeling and data cleaning: Assessment of atypical and extreme values ​​(`outliers´), detection and labeling of missing and/or non-applicable values, description of the distribution of each of the variables. Normality tests, scatter plots.

Basal comparative analysis: Comparison between the sociodemographic characteristics between the intervention group and the control group.

Final comparative analysis: The data will be analyzed based on the principle of intention to treat and adjusted by cluster.

Comparison of the clinical characteristics, between the control and intervention groups, using the t-test and chi-square test for cluster. If the assumptions of normality are not met, Somer's D test will be applied. The clinical relevance of the intervention will be determined from the dichotomous variable greater than 10% reduction in DHD with respect to the baseline estimate, the relative risk reduction (RRR), the absolute risk reduction (ARR) and the required number of physicians who need to be trained to achieve a relevant reduction in the prescription of benzodiazepines (NNT). A crude analysis adjusted for baseline characteristics (using "Generalized estimating equation") will be performed to determine the effect of belonging to the control or intervention group.

Subgroup analysis: The interaction term will be tested by introducing in the final GEE model, the interaction variable between arm and percentage of women, patients over 65 years of age and GP characteristics ( sex and working years).

**DATA ExtracTION**

Data will be extracted from three sources of information, the data on feasibility, acceptability, fidelity and adoption in the intervention group and the independent variables of the GPs of both groups, the data of DHD by GP extracted from the electronic prescription database of each Region, this data will have previously been agreed with the Medical Heads of each Region, monthly evolution data will be requested during the 12 months after the intervention by each GP, the third source of information comes from the electronic prescription system, the percentage of patients with chronic daily consumption in the 6 months prior to the intervention and at the end

In the research unit, the encryption will be unified and the 3 databases will be joined, a proportion of the data not less than 10% will be verified for its veracity with a double system of requesting and extracting data from a subsample of doctors and patients. . Once the verification tasks have been carried out, the data will be analyzed.

2 backup copies will be made on magnetic tape of all data entered each month (IBM 3500 backup server 400 Gb). The clinical information security office of each CCAA will ensure compliance with data protection regulations.

**LimitaTIONS OF THE STUDY**

There are few randomized studies evaluating the efficacy of an educational intervention in reducing the prescription of benzodiacepines. In this study we have used a methodology typical of implementation studies, with evaluation using pooled data extracted from electronic medication prescription databases, randomization at the health center level, and considering physicians as study subjects.

One of the most frequent limitations of prospective randomized clinical trials is the selection bias associated with loss to follow-up, on the other hand cluster randomized prospective clinical trials are also susceptible to selection bias in the inclusion of patients. The main advantage of adopting this methodology is precisely the absence of selection bias, since all patients are analyzed of each doctor and all the doctors of a health center are included before being randomized.

We evaluate the effectiveness through grouped data, which will allow us to evaluate the effectiveness of an intervention similar conditions of real conditions, as a brief training intervention could be easily applied in the PHCs. However, we consider that the main limitation of our study is the contamination that could occur between GPs themselves. If the intervention were clearly effective, the GPs could directly or indirectly induce the control group GPs to apply the intervention.

**ETHICAL ASPECTS**

Before carrying out the study, approval of the study was requested from the ethics committees of the Balearic Islands, CEI IDIAP Jordi Gol and CEI autonomous Primary Care of Valencia. The main researcher i will ensure that this study is carried out in accordance with the principles of the Declaration of Helsinki. ICH Good Clinical Practice (ICH/GCP) guidelines and in full compliance with relevant legal authorities. The protocol and all the required documents will be submitted for evaluation by the Ethics and Research Committee (CEI) and the Primary Care Research Commission. In addition, all substantial changes to the original documents will also be sent for evaluation and approval to the CEI and the competent authorities.

The trial will be registered on the website www.clinicaltrials.org.

This study will adhere to the CONSORT clinical trial registration guidelines. All participating researchers will receive at least one session of training and homogenization criteria and in these sessions, the dissemination and understanding of the same will be insisted on. It will be submitted to the Research Ethics Committee of the Balearic Islands and to the investigation commission of the Primary Care Management of Mallorca.

**SUBJECT CONFIDENTIALITY**

The sponsor and the researchers of the study will guarantee the confidentiality of the data of the subjects and will ensure that the provisions of Organic Law 15/1999 on the Protection of Personal Data are complied with at all times.

The processing of the data that the sponsor collects during the study will be subject to current legislation regarding data protection. The data of the participants will be anonymized, and the records will be identified only with an autonumeric code.

The trial staff will ensure that the anonymity of the participants is maintained. The GPs of the PHC that participate in the trial will be identified only by an identification number.

All documents will be stored securely and will only be accessed by trial staff and authorized personnel.

The clinical information security office of each Region will ensure compliance with data protection regulations. The data related to the prescription of benzodiazepines will be obtained grouped and anonymized with respect to the GP, at any time will we have any personal data of the patient or the doctor.

The investigator must keep copies of all documentation related to the clinical trial, for at least two years from the date of formal completion of the trial development. It is planned to keep these documents in the investigator's file. However, if the investigator is unable to comply with this obligation, he must request permission from the sponsor to have alternative measures available. Details of these measures will be documented.

**CONFLICT OF INTERESTS**

Investigators and staff at centers participating in the study should provide a full statement of those circumstances in which professional judgment about a primary concern, such as patient safety, financial benefit or research validity, may have any influence in the study.

**FINANCING AND INSURANCE**

This study has obtained funding from the Carlos III Health Institute.

**REFERENCES**

1.BNF 65 (2013): British National Formulary. 65th edn. London: British Medical Association and Royal Pharmaceutical Society of Great Britain.

2.Ministerio de Sanidad y Consumo. Guía de Prescripción Terapéutica (GPT). Adaptación española de la 51º ed. del British Nacional Formulary (BNF), 1º ed. Española. Barcelona: Pharma Editores S.L, 2006.

3.National Institute for Health and Clinical Excellence (NICE). Guidance on the use of zaleplon, zolpidem and zopiclone for the shortterm management of insomnia. [Internet]. London: National Institute for Health and Clinical Excellence (NICE). Technology Appraisal Guidance 77; [2004] [consultado el 15 marzo 2015]. Disponible en: http://www.nice.org.uk/nicemedia/live/11530/32845/32845.pdf.

4.Khong TP, de Vries F, Goldenberg JS, Klungel OH, Robinson NJ, Ibáñez L, Petri H. Potential impact of benzodiazepine use on the rate of hip fractures in five large European countries and the United States. Calcif Tissue Int 2012; 91:24-31.

5.Barbone F, McMahon AD, Davey PG, Morris AD, Reid IC, McDevitt DG et al. Association of roadtraffic accidents with benzodiazepine use. Lancet 1998;352(9137):1331-6.

6.Billioti de Gage S, Begaud B, Bazin F, Verdoux H, Dartigues JF, Peres K, et al. Benzodiazepine use and risk of dementia: prospective population based study. BMJ 2012; 345:e6231.

7.Billioti de Gage Sophie, Moride Yola, Ducruet Thierry, Kurth Tobias, Verdoux Hélène, Tournier Marie et al. Benzodiazepine use and risk of Alzheimer's disease: case-control study BMJ 2014; 349 :g5205

8.Belleville G. Mortality hazard associated with anxiolytic and hypnotic drug use in the National Population Health Survey. Can J Psychiatry 2010; 55(9):558-567.

9.Kripke DF, Langer RD, Kline LE. Hypnotics' association with mortality or cancer: a matched cohort study. BMJ Open 2012; 2: e000850.

10.Weich S, Pearce HL, Croft P, Singh S, Crome I, Bashford J, Frisher M. Effect of anxiolytic and hypnotic drug prescriptions on mortality hazards: retrospective cohort study. BMJ 2014; 348:g1996

11.American Geriatrics Society Beers Criteria Update Expert Panel. American GeriatrSociety Beers Criteria for potentially inappropiate medication use in older adults. J Am Geriatr Soc 2012; 24:731-8

12.Grupo de Trabajo de la Guía de Práctica Clínica para el Manejo de Pacientes con Trastornos de Ansiedad en Atención Primaria. Madrid: Plan Nacional para el SNS del MSC. Unidad de Evaluación de Tecnologías Sanitarias. Agencia Laín Entralgo. Comunidad de Madrid; 2008. Guías de Práctica Clínica en el SNS: UETS Nº 2006/10.

13.Grupo de Trabajo de la Guía de Práctica Clínica para el Manejo de Pacientes con Insomnio en Atención Primaria. Guía de Práctica Clínica para el Manejo de Pacientes con Insomnio en Atención Primaria. Plan de Calidad para el Sistema Nacional de Salud del Ministerio de Sanidad y Política Social. Unidad de Evaluación de Tecnologías Sanitarias. Agencia Laín Entralgo. Comunidad de Madrid; 2009. Guías de Práctica Clínica en el SNS: UETS Nº 2007/5-1.

14.Agence française de securité sanitaire des produits de santé (afssaps). Etat des lieux de la consommation des benzodiazepines en France [monografía en internet]. Saint Denis (Paris): [2013] [consultado el 15 mar de 2015]. Disponible en: <http://ansm.sante.fr/> content/download/57511/738785/version/2/file/ANSM_Rapport+Benzo_09012014.pdf

15.Agencia Española de Medicamentos y Productos Sanitarios. Observatorio Uso de Medicamentos. Utilización de medicamentos ansiolíticos e hipnóticos en España durante el período 2000-2012 [Internet]. Madrid: AEMPS; [2014] [consultado el 15 mar 2015]. Disponible en: http://www.aemps.gob.es/medicamentosUsoHumano/observatorio/docs/ansioliticos_hipnoticos-2000-2012.pdf

16.Bejarano F, Pinol JL, Mora N, Claver P, Brull N, Basora J. Elevado consumo de benzodiacepinas en mujeres ancianas asignadas a centros de salud urbanos de atención primaria. Aten Primaria 2008; 40:617-621.

17.Vicens C, Fiol F, Llobera J, Campoamor F, Mateu C, Alegret S, Socías I. Withdrawal from long-term benzodiazepine use: randomised trial in family practice. Br J Gen Pract 2006; 56(533):958-963.

18.Sirdifield C, Anthierens S, Creupelandt H, Chipchase SY, Christiaens T, Siriwardena AN. General practitioner's experiences and perceptions of benzodiazepine prescribing: Systematyc review and meta-synthesis. BMC Family Practice 2013; 14:191

19.Voshaar RC, Couvee JE, van Balkom AJ, Mulder PG, Zitman FG. Strategies for discontinuing long-term benzodiazepine use: meta-analysis. Br J Psychiatry 2006; 189:213-220.

20.Parr JM, Kavanagh DJ, Cahill L, Mitchell G, McD Young R. Effectiveness of current treatment approaches for benzodiazepine discontinuation: a meta-analysis. Addiction 2009; 104(1):13-24.

21.Gould R, Coulson MC, Patel N, Highton-Williamson E Howard R. Interventions for reducing benzodiazepine use in older people: meta-analysis of randomised controlled trials. Br Psychiatry 2014; 204 (2) 98-107; DOI: 10.1192/bjp.bp.113.126003

22.Vicens C, Bejarano F, Sempere E, Mateu C, Fiol F, Socias I, Aragonès E, et al. Comparative efficacy of two interventions to discontinue long-term benzodiazepine use: a cluster randomised controlled trial in primary care. Br J Psychiatry 2014. doi:10.1192/ bjp.bp.113.134650.
