# Supplementary material for: Evaluation of a multicomponent intervention consisting of education and feedback to reduce benzodiazepine prescriptions by general practitioners: The BENZORED hybrid type 1 cluster randomized controlled trial
Source: PLoS Med. 2022 May 6;19(5):e1003983. doi: 10.1371/journal.pmed.1003983 (PMC9075619; doi:10.1371/journal.pmed.1003983)
Supplement: S1 Analysis plan — (DOCX) [file pmed.1003983.s006.docx]

*Predefinied analysis plan*

Estimated effects will be calculated by comparing the DHDs of GPs in the intervention and control groups at 12 months. A generalised mixed linear random effect models will be used to account for clustering at the level of the healthcare centre, and adjusted for baseline values of the outcome measures. All analyses will be performed on an ‘intention-to-treat’ basis (ie, all initially enrolled GPs will be included in the analysis according to the group to which they were assigned) and will be reported according to 2010 Consolidated Standards of Reporting Trials guidelines.

Subgroup analysis of effectiveness by regions will also be performed. We will also test for significant differences in the baseline characteristics of the control and intervention groups using descriptive analysis. This will include calculation of means and/or proportions with CIs, and on robusts SDs (to account for clustering). We will test for differences in the implementation success by descriptive analysis of the CFIR constructs. .

Amendments to the analysis plan

Initially, the use of generalized mixed linear random effect models was planned to compare data for the primary outcome. However, a change was made in the statistical plan.  We carried out a mixed effects Poisson regression model to account for the distribution of the DDDs per 1000 inhabitants-per-day of BZDs

Date of Final Statistical Analysis Plan: 07/01/2016 Amendment No. 1: 23/08/2021.
