# Supplementary material for: Evaluation of a multicomponent intervention consisting of education and feedback to reduce benzodiazepine prescriptions by general practitioners: The BENZORED hybrid type 1 cluster randomized controlled trial
Source: PLoS Med. 2022 May 6;19(5):e1003983. doi: 10.1371/journal.pmed.1003983 (PMC9075619; doi:10.1371/journal.pmed.1003983)
Supplement: S1 Questionnaire — Six items fidelity questionnaire. (PDF) [file pmed.1003983.s007.pdf]

8076592063

Id Benzored study | | | |

Dear Colleague,

We would like to know your opinion about the BENZORED research project in which you have participated. In order to evaluate the implementation of the intervention, we invite you to answer a series of questions:
This survey is anonymous, all the information you provide us will be treated as strictly confidential and will form part of the global analysis of all surveys.
It will take approximately 15 minutes to complete the questionnaire.

Thank you very much for your cooperation.

Please circle the answer option that best fits your own opinion:
Exemple

1 2 3 4 5 6 7 8 9 10

**Initial prescription**	Completely disagree									Completely agree
1. I discussed benefits and risk with the patient before the first prescription?	1	2	3	4	5	6	7	8	9	10
2. I limited the duration of the initial prescription from the start of the treatment	1	2	3	4	5	6	7	8	9	10
**Benzodicepine withdrawal in long-term user**	Completely disagree									Completely agree
3. I identified long-termuseras and use the tailoring stepped dose reduction to discontinuing BZD use in long-term users	1	2	3	4	5	6	7	8	9	10
**Access to the Benzored Web page**	Completely disagree									Completely agree
4. I accessed the benzored web page	1	2	3	4	5	6	7	8	9	10
5. I downloaded and delivered the patient information leaflet about BZDs, Z-drugs, and sleep hygiene	1	2	3	4	5	6	7	8	9	10
**Graphical information about BZD prescription**	Completely disagree									Completely agree
6. I received and revised my monthly feed-back about BZD prescription	1	2	3	4	5	6	7	8	9	10
