# Supplementary material for: Evaluation of a multicomponent intervention consisting of education and feedback to reduce benzodiazepine prescriptions by general practitioners: The BENZORED hybrid type 1 cluster randomized controlled trial
Source: PLoS Med. 2022 May 6;19(5):e1003983. doi: 10.1371/journal.pmed.1003983 (PMC9075619; doi:10.1371/journal.pmed.1003983)
Supplement: Attachment — Submitted filename: Carta al editor con cambios ACEPTADOS CV.docx [file pmed.1003983.s008.docx]

Dear Caitlin Moyer (Associate Editor)

We highly appreciate the detailed and valuable comments of the academic editor and referees on our manuscript, “Effectiveness of a multicomponent intervention consisting of education and feedback on reducing benzodiazepine prescriptions by general practitioners: BENZORED hybrid type 1 cluster randomized controlled trial” (PMEDICINE-D-21-03642R2). We provided point-by-point replies to all of these comments below and carefully revised the manuscript accordingly.

Thank you very much for your time and interest.

We look forward to hearing from you soon.

Sincerely,

Alfonso Leiva

Requests from the editors:

**From the Academic Editor:**

1. Please include a description of the intervention, using the TIDIER checklist or equivalent as a guide.

**Response:** Thank you for providing us the opportunity to address the comments from the reviewers and the academic editor. We carefully considered all feedback, and hope that all revisions to the manuscript are satisfactory.

We expanded this section as suggested by the academic editor (points #17 and #22) and reviewer #1. We included the TIDieR checklist.

The multicomponent BENZORED intervention consisted of an educational meeting for GPs, audit and feedback, and a support web page.

Educational meetings are commonly used for continuing medical education with the aim of improving professional practice and patient outcomes. Educational meetings include courses, conferences, lectures, workshops, seminars, and symposia.[40]

At the start of the trial, the GPs from the intervention group received a 2-hour educational face to face workshop in their PHCs. These interventions were delivered by researchers who were GPs, had great expertise in prescribing and deprescribing BZD, and provided training about appropriate procedures for prescribing BZDs. This included educational information about the pharmacological properties of BZDs (biological half-life and equivalent dose of different BZDs), indications for prescription, recommended duration of use, adverse effects, dependence, tolerance, prevalence, and consequences of long-term use. These GPs were asked to discuss with their patients harms and benefits of taking BZD before the first prescription. They received training in a structured BZD discontinuation intervention based on gradual BZD dose reduction and training based on real clinical cases. [1,33]

After the initial training workshop, all participating GPs received automated monthly feedback. The audit and feedback, based on the “Feedback Intervention Theory”[45], provided a summary of the clinical performance of health care over a specified period of time that was aimed at changing the practices of health professional.[41]

Graphical depictions of BZD prescription feed-back consisted of a line graph that plotted each GP’s monthly BZD prescription rate in DDDs per 1000 inhabitants per day for 12 months, and two additional line graphs that plotted the BZD prescription rate of the PHC and the health district. The GPs also received a password to access to a support web page that provided additional information to reinforce the messages of the workshop. This web site included videos, descriptions of practical cases, and an information leaflet for patients about BZDs, Z-drugs, and sleep hygiene (http://benzored.es).

2. Please include the CONSORT checklist for cluster randomized trials. AL
**Response:** We included the CONSORT checklist.

3. Please describe the intra-cluster correlation coefficient in the Results section and please clarify if this was lower than originally assumed for the sample size calculation.

**Response:** We thank the academic editor for highlighting this point. We included this additional information in the Results as follows:

“At the PHC level, the intra-cluster correlation coefficient of DDDs per 1000 inhabitants per day of BZDs was 0.30. This was higher than expected from the sample size calculation, indicating substantial within-PHC clustering for BZD prescriptions.”

Other editorial points:

4. Data availability statement: PLOS Medicine requires that the de-identified data underlying the specific results in a published article be made available, without restrictions on access, in a public repository or as Supporting Information at the time of article publication, provided it is legal and ethical to do so. Please see the policy at
<http://journals.plos.org/plosmedicine/s/data-availability> and FAQs at
<http://journals.plos.org/plosmedicine/s/data-availability#loc-faqs-for-data-policy>

Thank you for your willingness to make your data available in a repository. However, the file “repository.sav” appears to be a proprietary format (SPSS dataset) that may reduce the accessibility. We request that authors provide data in file formats that are standard in their field and allow wide dissemination. If there are currently no standards in the field, authors should maximize the accessibility and reusability of the data by selecting a file format from which data can be efficiently extracted.

**Response:**We uploaded a new version of the dataset in a spreadsheet. 10.521/zenodo.5607352

5. Throughout text: Please include line numbers with the revised version.

**Response:**Thanks, we included line numbers in the manuscript.

6. Abstract: Please report your abstract according to CONSORT for abstracts, following the PLOS Medicine abstract structure (Background, Methods and Findings, Conclusions) <http://www.consort-statement.org/extensions?ContentWidgetId=562>

**Response:**As suggested, we revised the Abstract according to the CONSORT statements for abstracts as follows:

Background: Current benzodiazepines (BZD) prescription guidelines recommend short-term use to minimize the risk of dependence, cognitive impairment, and falls and fractures. However, many clinicians overprescribe BZDs and chronic use by patients is common. There is limited evidence on the effectiveness of interventions delivered by GPs on reducing prescriptions and long-term use of BZDs.

Objective: To evaluate the effectiveness of a multi-component intervention for general practitioners (GPs) that seeks to reduce BZD prescriptions and the prevalence of long-term users.

Methods and Findings: We conducted a multicenter two-arm, cluster randomized controlled trial in three health districts in Spain (primary health centers [PHC] in Balearic Islands, Catalonia, and Valencian Community) from September 2016 to May 2018. The 81PHCs were randomly allocated to the intervention group (n= 41; 372 GPs) or the control group (n= 40; 377 GPs). GPs were not blinded to the allocation. The intervention consisted of a workshop about the appropriate prescribing of BZDs and withdrawal using a tailored stepped dose reduction with monthly BZD prescription feedback and a support web page. The primary outcome, based on 700 GPs (351 in the control group and349 in the intervention group), compare changes in BZD prescriptions in defined daily doses (DDD) per 1000 inhabitants per day after 12 months. The two secondary outcomes were the proportion of long-term users and proportion of long-term users over age 65 years. The intervention and control groups were not homogeneus in some characteristics.

After 12 months there were a statistically significant decline in total BZD prescription in the intervention group compared to the control group (mean difference: −3.24 DDDs per 1000 inhabitants per day, 95% Confidence Interval: −4.96, –1.53, p<0.001). The intervention group also had a smaller number of adjusted relative long-term users overall (-3,6% (95%CI: 1.85,8.88, p>0,001) and a smaller number of adjusted relative long-term users over age 65 years (.- 3.5% (95%CI: 1.19, 5.71 p=0,004).

Conclusions: A multicomponent intervention that targeted GPs and included educational meeting, feedback about BZD prescriptions, and a support web page led to a statistically significant reduction of BZD prescriptions and fewer long-term users.   Although the effect size was small, the high prevalence of BZD use in the general population suggests that large-scale implementationof  this intervention could have positive effects on the health of many patients.

Trial registration: ISCRTN ISRCTN28272199

Funding: Carlos III Institute, Ministry of Economy and Competitiveness.

7. Abstract: Please specify who was blinded to the intervention and control, and please mention any GPs or PHCs that were lost to follow up in each group.

**Response:** Thanks, we added this information to the Methods and Findings section of the Abstract (above).

8. Abstract: Methods and Findings: Please quantify the main results for decline in BZD prescriptions and proportions of long-term users over age 65 including both 95% CIs and p values. Please present findings for long term users in general.

**Response:** Thanks, we added this information to the Methods and Findings section of the Abstract (above).

9. Abstract: Please report a summary of any adverse events in the Abstract (and please also present these findings in the Results of the manuscript).

**Response:** Data were aggregated at the GP level. We did not collect any individual information of patients related to adverse events.

10. Abstract: In the last sentence of the Abstract Methods and Findings section, please describe the main limitation(s) of the study's methodology.

**Response:** Thanks, we added this information to the Methods and Findings section of the Abstract (above).

11. Author summary: At this stage, we ask that you include a short, non-technical

Author Summary of your research to make findings accessible to a wide audience that

includes both scientists and non-scientists. The Author Summary should immediately

follow the Abstract in your revised manuscript. This text is subject to editorial change

and should be distinct from the scientific abstract. Please see our author guidelines for

more information:

[https://journals.plos.org/plosmedicine/s/revising-yourmanuscript#loc author-summary](https://journals.plos.org/plosmedicine/s/revising-your-manuscript#loc-author-summary)

**Response:**As suggested, we added a nontechnical author summary in the manuscriptas follows:

“Why Was This Study Done?

- Long-term use of benzodiazepines is common, even though prescription guidelines recommend limiting treatment to a few weeks.
- There is some evidence that interventions implemented by GPs can reduce long-term prescriptions of benzodiazepines.
- The evidence supporting the effectiveness of these interventions is limited.

What Did the Researchers Do and Find?

A controlled cluster randomized clinical trial was conducted in 81 PHCs in three regions of Spain to examine the impact of a multicomponent strategy on reducing benzodiazepine prescriptions.

- The multicomponent intervention targeted GPs and was based on a 2-h educational workshop, audit and feedback about prescription practices, and access to a support webpage.
- At follow-up, the intervention group had a statistically significant declines in total benzodiazepine prescriptions (−3.24 DDDs per 1000 inhabitants per day) and in the percentage of long-term users (−3.6%).

What Do These Findings Mean?

- Implementing a brief multicomponent intervention that targets GPs can reduce benzodiazepine prescriptions.
- A tailored stepped-dose reduction for discontinuing benzodiazepines successfully reduced the proportion of long-term users and may also reduce adverse outcomes related to long-term use.”

12. Main text: For citations within the text, please include the reference number within square brackets, placed before the sentence punctuation. Where multiple references are indicated, please do not include spaces within the brackets.

**Response: Agreed**

13. Methods: Please include the study protocol/predefined analysis plan, with any amendments, as Supporting Information.

**Response:**We thank the academic editor for highlighting this point. The analysis plan was in the protocol, and we added an amendment to the statistical analysis section in the attached supporting information as follows:

“Initially, the use of generalized mixed linear random effect models was planned to compare the primary outcome in the two groups. However, this was changed to a mixed effects Poisson regression model to account for the distribution of theDDDs per 1000 inhabitants per day of BZDs.”

14. Methods: Please describe the nature of GP consent to participate.

**Response:** We thank the editor for this comment. The head of the PHC signed the written consent document for participation. This consent indicated that all GPs were informed about the study and allowed the researchers to receive emails and use their prescription databases for the study. The Balearic Islands Ethical Committee of Clinical Research (IB3065/15), l'IDIAP Jordi Gol Ethical Committee of Clinical Research (PI 15/0148), and the Valencia Primary Care Ethical Committee of Clinical Research (P16/024) classified this study as a “service development/audit” and did not require individual GP consent. We added the following text:

“All GPs from participating PHCs were included and primary and secondary outcomes for analysis were at the GP level.”

15. Methods: Study population: Please mention the three regions are part of Spain.

**Response:**We included this information in the study population section as follows:

“PHCs from the following regions of Spain were eligible for participation: Balearic Islands (IbSalut), Catalonia (InstitutCatalà de la Salut; Tarragona-Reus district), and Valencian Community (Conselleria de Salut Universal; Arnau de Vilanova-Llíria district).”

16. Methods: Please complete the CONSORT checklist and ensure that all components of CONSORT are present in the manuscript.

When completing the checklist, please use section and paragraph numbers, rather than page numbers.

**Response:**We attached the CONSORT checklist, and carefully ensured that all components were present in the manuscript

In addition to the CONSORT checklist, we suggest that your implementation research be reported according to Standards for Reporting Implementation Studies statement (STARI). The STARI guidelines can be found here: <https://www.equator-network.org/reporting-guidelines/stari-statement/>

**Response:**We attached the STARI check list.

17. Methods: Please describe the intervention (educational workshop, training on discontinuation/dose reduction intervention, automated monthly feedback, support web page) in greater detail.

**Response:**As detailed above, we expanded the intervention section as follows:

The multicomponent BENZORED intervention consisted of an educational meeting for GPs, audit and feedback, and a support web page.

Educational meetings are commonly used for continuing medical education with the aim of improving professional practice and patient outcomes. Educational meetings include courses, conferences, lectures, workshops, seminars, and symposia.[40]

At the start of the trial, the GPs from the intervention group received a 2-hour educational face to face workshop in their PHCs. These interventions were delivered by researchers who were GPs, had great expertise in prescribing and deprescribing BZD, and provided training about appropriate procedures for prescribing BZDs. This included educational information about the pharmacological properties of BZDs (biological half-life and equivalent dose of different BZDs), indications for prescription, recommended duration of use, adverse effects, dependence, tolerance, prevalence, and consequences of long-term use. These GPs were asked to discuss with their patients harms and benefits of taking BZD before the first prescription. They received training in a structured BZD discontinuation intervention based on gradual BZD dose reduction and training based on real clinical cases.[1,33]

After the initial training workshop, all participating GPs received automated monthly feedback. The audit and feedback, based on the “Feedback Intervention Theory”[45], provided a summary of the clinical performance of health care over a specified period of time that was aimed at changing the practices of health professional.[41]

Graphical depictions of BZD prescription feed-back consisted of a line graph that plotted each GP’s monthly BZD prescription rate in DDDs per 1000 inhabitants per day for 12 months, and two additional line graphs that plotted the BZD prescription rate of the PHC and the health district. The GPs also received a password to access to a support web page that provided additional information to reinforce the messages of the workshop. This web site included videos, descriptions of practical cases, and an information leaflet for patients about BZDs, Z-drugs, and sleep hygiene (http://benzored.es).

18. Methods: Please provide more description of how the DDD per 1000 inhabitants per day was calculated.

**Response:**To provide clarification of defined daily dose (DDD), we added the following text :

“The primary outcome measure was DDDs of BZDs per 1000 inhabitants per day after 12 months. The total number of dispensed DDDs from the N05BA, N05CD, and N05CF groups of the Anatomical Therapeutic Chemical (ATC) Classification System code was extracted from the e-prescription database of each health district. Total number of dispensed DDD was divided by total GP’s patient list /1000 x 365 days.

19. Methods: Please provide more description of the prescriptions claims databases used to extract prescription records.

**Response:**Thank you for highlighting the need for this information. We added the following text:

“The RELE (Ibsalut, Balearic Islands), Rec@p (InstitutCatalà de la Salut; Tarragona-Reus District), and Recetaelectrónica (Conselleria de Salut Universal; Arnau de Vilanova-Llíria District) are the e-prescription systems and databases used in the three health districts included in this study.

These databases contain information about all primary care prescriptions dispensed in community pharmacies, including dispensing date, pharmaceutical product (active ingredient and brand name), dose, and treatment duration.”

20. Methods: Please provide additional description of the subgroup analyses.

**Response:**We extended the description of the subgroup analysis as follows:

“To assess whether the effect of the intervention on BZD prescription varied according to health district, a subgroup analysis of effectiveness that was previously planned in the protocol was performed”

21. Methods: The trial registration lists the outcomes of “3. Feasibility, adoption and fidelity of the intervention will be measured by an "ad hoc" questionnaire to measure GP opinion”. (a) Can you please present those results as part of this manuscript, or indicate why that is not possible? (b) Can you please indicate when you plan to publish those results?

**Response:** We appreciate the opportunity to clarify this important point. We acknowledge that evaluation of the implementation process is an important consideration in evaluation of the intervention. We therefore included a brief summary of methods and findings. However, there were some changes from the planned evaluation of the implementation process. We changed to a CFIR model because this framework also considers factors at the organizational level. We revised the text as follows:

“Implementation process.

Full details of the methods used for evaluation of the implementation of the BENZORED IV intervention were reported elsewhere.[49] To summarize, 40 semi-structured interviews and 5 focus group meetings were conducted. GPs were invited to participate in an effort to ensure representativeness for the different health district locations, and they were classified as ‘low prescribers’ or ‘high prescribers’ based on the 12-month final evaluation.

The Consolidated Framework Implementation Research (CFIR) was used to guide development of the focus group meetings and for coding and data analysis. [42] The CFIR is a theoretical framework that provides a list of 41 constructs organized in five domains that can negatively or positively influence implementation.

Among the 45 GPs contacted, 40 agreed to interviews.

The implementation fidelity was measured as adherence to the intervention, defined as whether "a program service or intervention is being delivered as it was designed". [50] Adherence was measured using a questionnaire given to all GPS in the intervention group. This questionnaire asked the GPs to rank adherence to each of the following components of the intervention on a scale from 1 to 10: discussing benefits and risk with the patient before the first prescription; setting limits for the duration from the start of treatment; tailoring stepped dose reduction to discontinue BZD use in long-term users; reviewing BZD prescription feedback; downloading and using the patient information leaflet about BZDs, Z-drugs, and sleep hygiene; and visiting the web page to reinforce information.

**Result section**

Evaluating the implementation process

A total of 246/372 (66%) GPs from the intervention group answered this questionnaire. These GPs generally valued the multicomponent intervention as flexible and integrable into their practices. However, the three components of the intervention were valued differently. They reported the educational workshop was useful for clinical practice and the BZD prescription feedback was easy to interpret. They reported the tailored stepped dose reduction regimen for discontinuing BZDs was complex, and their lack of time and workload were significant barriers to implementation. The material included in the web page was considered helpful for the patients, but they reported some technical problems in accessing the website.

The fidelity of adherence to the intervention differed among the different components. Discussing benefits and risk with the patient before the first prescription had a median adherence score of 8 (interquartile range [IQR]: 7 to 9); setting limits for the duration from the start of the treatment had a median score of 8 (IQR: 7 to 9); tailoring stepped dose reduction to discontinuing BZD use in long-term users had a median score of 7 (IQR: 7 to 8 ); reviewing BZD prescription feedback had a median score of 7 (IQR: 6 to 8); downloading and using the patient information leaflet about BZDs, Z-drugs, and sleep hygiene had a median score of 7 (IQR: 7 to 8); and visiting the web page to reinforce information had a median score of 5 (IQR: 3.75 to 7).

22. Methods: Please provide more detail on the implementation aspect of the trial. Please clearly define and differentiate and define your intervention and implementation strategy and outcomes for each. It should be clear how both aspects were tested for this hybrid trial. As described, the multicomponent interventions seem more like implementation strategies.

Please consider using the guidelines published by Proctor et al. to guide your reporting: Proctor EK, Powell BJ, McMillen JC: Implementation strategies: recommendations for specifying and reporting. Implement Sci 2013, 8:139
Please ensure you have included relevant and sensitive implementation outcomes to capture the impact of individual, team-level, and organizational factors that could determine the success/failure of the intervention. Example document on Implementation outcomes. Please use standard terminology for implementation outcomes so that there is consistency in reporting (e.g. adoption, fidelity, penetration, sustainability etc.).

Please clarify whether you used suitable implementation theoretical frameworks to guide the design of the study, analysis, and interpretation of your findings.

**Response:** We thank the reviewer for highlighting this point, and for the opportunity to provide further clarification on the implementation of the trial. We included a brief summary of methods and findings of the implementation process (point #21). In particular, weincluded recommendations for specifying and reporting of the intervention as the reviewer suggested:

The multicomponent BENZORED intervention consisted of an educational meeting for GPs, audit and feedback, and a support web page.

Educational meetings are commonly used for continuing medical education with the aim of improving professional practice and patient outcomes. Educational meetings include courses, conferences, lectures, workshops, seminars, and symposia.[40]

At the start of the trial, the GPs from the intervention group received a 2-hour educational face to face workshop in their PHCs. These interventions were delivered by researchers who were GPs, had great expertise in prescribing and deprescribing BZD, and provided training about appropriate procedures for prescribing BZDs. This included educational information about the pharmacological properties of BZDs (biological half-life and equivalent dose of different BZDs), indications for prescription, recommended duration of use, adverse effects, dependence, tolerance, prevalence, and consequences of long-term use. These GPs were asked to discuss with their patients harms and benefits of taking BZD before the first prescription. They received training in a structured BZD discontinuation intervention based on gradual BZD dose reduction and training based on real clinical cases.[1,33]

After the initial training workshop, all participating GPs received automated monthly feedback. The audit and feedback, based on the “Feedback Intervention Theory”[45], provided a summary of the clinical performance of health care over a specified period of time that was aimed at changing the practices of health professional.[41]

Graphical depictions of BZD prescription feed-back consisted of a line graph that plotted each GP’s monthly BZD prescription rate in DDDs per 1000 inhabitants per day for 12 months, and two additional line graphs that plotted the BZD prescription rate of the PHC and the health district. The GPs also received a password to access to a support web page that provided additional information to reinforce the messages of the workshop. This web site included videos, descriptions of practical cases, and an information leaflet for patients about BZDs, Z-drugs, and sleep hygiene (http://benzored.es).

23. Results: The sample size listed in the submitted manuscript (749 GPs) and the target from the trial registry (508 GPs) differ. Please explain the discrepancy.

**Response:** Thanks for this comment. We increased the sample size to increase our ability to detect clinically meaningful differences.

24. Results: Please provide 95% CIs and p values for all primary and secondary outcomes and subgroup analyses described in the text. For the subgroup analyses, please report the interaction between health district and outcome. When reporting adjusted analyses, please also mention the factors adjusted for.

**Response:As suggested, w**e included 95% CIs and p values for all primary and secondary outcomes and subgroup analyses. Please note that we reported absolute and relative reduction for the percentage of BZD long-term users but only reported p values for absolute values, because relative reduction was calculated as the percentage change from baseline.

**We included the interaction term and added the following text:**

**“The interaction term from DDDs per 1000 inhabitants per day and group was statistically significant among health districts (p = 0.002).”**

We also reported the factors adjusted for in the multivariate analysis.

**“Table 2. Comparison of General Practitioners’ benzodiazepine prescriptions and long-term users. Results were unadjusted or adjusted by DDD per 1000 inhabitants per day and percentage of BZD long-term users at baseline for the control and intervention groups at the 12-month follow-up.”**

25. Results: Please report any findings pertaining to adverse events for the study including numbers of specific events and whether or not adverse events are thought to be related to the intervention.

**Response:**The data used for the graphical audit and feedback and to evaluate the effectiveness of the intervention were retrospectively collected from the prescription database. We did not collect any information regarding adverse events in individual patients.

26. Discussion: Please present and organize the Discussion as follows: a short, clear summary of the article's findings; what the study adds to existing research and where and why the results may differ from previous research; strengths and limitations of the study; implications and next steps for research, clinical practice, and/or public policy; one-paragraph conclusion

Discussion

Our major finding is that a multicomponent intervention that targeted GPs was effective in reducing prescriptions of BZDs and in reducing the proportion of long-term BZD users at twelve months.

Comparison with existing literature

Our study indicated a small reduction in the total number of BZD prescriptions. A previous study found greater reductions in the prescriptions of BZDs to elderly patients (measured as DDD per 1000 inhabitants per day) following continuous educational outreach visits to GPs. This previous study used a more intensive intervention that consisted of visits between GPs and pharmacists every 2 to 8 weeks.[51] However these more intensive interventions are more difficult to implement in most PHCs. In contrast, other authors found that educational visits with GPs regarding BZD prescriptions were not effective in reducing the number of prescriptions.[52,53] Pimlott et al. and Holm also found that an educational intervention and feedback to GPs was not effective.[54,55]

Changing inappropriate health interventions is essential for minimizing patient harm, maximizing efficient use of resources, and improving population health. Prescribing BZDs in patients aged 65 years or more is considered inappropriate and should be avoided. We found that our intervention led to a 3.5% reduction in the use of BZDs in long term users who were aged 65 years or more. There is evidence that certain interventions delivered by GPs were effective in reducing the number of long-term BZD users.[27] A previous study of a stepped-dose reduction of BZDs, with written instructions to long-term users delivered by GPs to long-term users, showed that the intervention was three-times more effective than usual care.[56] Similar results (25% reduction of BZD use by long-term users) were reported in an intervention consisting of sending an educational booklet and a component for risk self-assessment of BZD use to long-term users.[57] These results differ from our finding that the intervention implemented by GPs provided only a modest reduction of BZD use by long-term users. In more pragmatic clinical trials, GPs receive educational training, and all patients are evaluated, but not all patients receive the intervention. However, a small effect in a pragmatic trial that includes most GPs and all eligible patients is clinically relevant in absolute terms, because overuse of BZDs is widespread in Spain.

The overall reduction in BZD prescriptions that we observed in the intervention group was similar in magnitude to that found in an intervention that included education with audit and feedback.[40,41]

Our results indicated that the intervention led to a reduction of 3.2 DDD per 1000 inhabitants per day. From a health system perspective, this reduction over a 1-year period is clinically important. Some other health systems are steadily decreasing their prescriptions for BZDs. For example, Norway decreased BZD prescriptions from 65.7 to 49.7 DDD per 1000 inhabitants per day from 2008 to 2018. The decline in Denmark over the same period was 42.4 to 25.9 DDD per 1000 inhabitants per day. However, such decreases did not occur in Spain, Portugal, or France. In fact, over this same time, Spain had an increased consumption of BZDs from 75.5 to 89.3 DDD per 1000 inhabitants per day.[18]

Strengths and limitations

To our knowledge, this is the largest clinical trial to analyze the effectiveness of an educational intervention for GPs in a primary care setting that focused on reducing prescriptions for BZDs. We included all GPs of the participating PHCs. The study had a high rate of GP participation compared to similar studies[54], and very few GPs were lost to follow up. We also found differences in effect size among the different health districts, indicating that the characteristics of the health district affected the efficacy of the intervention. For example, the Balearic Islands and Tarragona have local policies regarding BZD prescribing that include indicators and incentives. Another strength of our study is that it was performed in three health districts that have different characteristics. This indicates the results are applicable in different clinical settings.

Our study had also some limitations. At baseline, the intervention group had more GP training healthcare centers and more GP trainers and physician doctors. This could have led to an overestimate of the effectiveness of the intervention because GP trainers and physician doctors are more likely to follow clinical guidelines and tend to write fewer BZD prescriptions. [21] Although we did not adjust for GP characteristics, we did adjust for baseline BZD prescriptions to minimize the potential of selection bias. GPs in the intervention PHCs may have shared information and strategies with GPs of the usual care PHCs. However, we randomized PHCs to avoid cross-contamination, and the PHCs were mostly in different villages or cities. Moreover, only GPs in the intervention arms had access to the additional material from the website and received individual BZD prescription feedback that was sent to their institutional e-mail addresses.

Implications for clinical practice and future research

A minimal intervention provided to GPs in a primary care setting, which consists of an educational training program and feedback regarding BZD prescriptions, may help to reduce the overall number of BZD prescriptions and reduce the number of long-term BZD users. From a public health perspective, because BZD use is very common in Spain, a small effect on long-term use by individuals could have a large impact in preventing adverse effects related to BZD use at the level of the population, such as falls, fractures, or cognitive impairment, especially in the elderly. In conclusion, our rigorous trial design and theory-based evaluation of the implementation process provided evidence of the effectiveness of a multicomponent strategy that targeted GPs to help reduce BZD prescriptions and the number of long-term users.

27. References: Please use the "Vancouver" style for reference formatting and see our website for other reference guidelines

https://journals.plos.org/plosmedicine/s/submission-guidelines#loc-references
**Response: Agreed**

28. Tables 1 and 2: Please include these in the main text of the manuscript.

**Response:** We included as suggested.

29. Table 2: Please note in the legend factors adjusted for in the analyses. Please also include results of unadjusted analyses.

**Response:** Thanks for the comment. We added the unadjusted analyses to Table 2 and added in the legend that the effect size was adjusted by DDD per 1000 inhabitants per day and percentage of BZD long-term users at baseline. We also included the unadjusted effect size.

“Table 2. Comparison of General Practitioners’ benzodiazepine prescriptions and long-term users. Results were unadjusted or adjusted by DDD per 1000 inhabitants per day and percentage of BZD long-term users at baseline for the control and intervention groups at the 12-month follow-up.”

30. Supporting Information Table 1: Please include p values for the subgroup analyses by health district, including the analysis for the interaction, for effectiveness of intervention within districts and comparisons between health districts.
**Response:** As suggested, we included p-values in the supplementary table and values for the interaction term in the manuscript.

**“The interaction term from DDDs per 1000 inhabitants per day and group was statistically significant (p = 0.002) among health districts.”**

**Comments from the reviewers:**

Thank you for the opportunity to address the comments of the reviewers. We hope that they will be satisfied with our revisions.

**Reviewer #1:**

In short: relevant topic, large well-conducted study, conventional intervention, concise presentation.
Abstract
I would recommend to describe the effect size as small, similar to the conclusion in the discussion section.

**Response:**We added the following text to the Abstract:

Conclusions: A multicomponent intervention that targeted GPs and included educational meetings, feedback about BZD prescriptions, and a support web page led to a statistically significant reduction of BZD prescriptions and fewer long-term users. Although the effect size was small, the high prevalence of BZD use in the general population suggests that large-scale implementation of this intervention could have positive effects on the health of many patients.

Introduction
It would be relevant to specify that the prescription rates for BZB in Spain are relatively high compared to other countries (information that I got from the discussion).

**Response:**As reported in the Introduction, we discuss the relatively high rates of BZD prescriptions in other countries:

“The prevalence of BZD use varies greatly among countries and ranges from less than 15 defined daily doses (DDDs) per 1000 inhabitants per day in the United Kingdom, the Netherlands, and Germany, to more than 85 DDDs per 1000 inhabitants per day in countries such as Iceland, Portugal, and Spain(18).”

The choice of the implementation strategies (education, feedback, and information platform) should be founded in preparatory research and/or conceptual analysis of current practice. Currently it remains unclear why exactly these strategies were chosen, which is not consistent with current implementation science.

Deprescribing (and de-implementation generally) has received considerable interest in recent years. However, this manuscript does not relate to the implementation science literature on the topic.

**Response:**Wethank the reviewer for highlighting this important point. We extended the Introduction to explain the most important implementation strategies that target professional behaviors and the most frequent deprescribing interventions:

To change the BZD prescribing behaviors of health professionals and taper BZD use by long-term users, it is important to reduce the risk of dependence and related adverse events from BZD use. The most common deprescribing interventions include the identifying appropriate patients for deprescribing, providing education and development training to the general practitioners and patients, and using tailored stepped-dose reduction of BZDs[35,36]. The most common implementation strategies are targeting professional behavioral changes by using printed educational materials, educational meetings, educational outreach, local opinion leaders, audit and feedback, computerized reminders, and tailored interventions [37–40]. Audit and feedback and educational meetings are widely used in clinical practice as quality improvement measures. Two systematic reviews that examined 220 randomized controlled trials showed these had a small to moderate effect on changing the behaviors of health professionals. However, there was wide variation in their impact and the extent to which they were implemented.[40,41]

The chosen approach is conventional for the implementation of recommended practices, although well-conducted at large scale. The authors argue that the prevention of first prescription of BZB has rarely been studied. This may strictly be true, but not prescribing has certainly be a topic in other medication.

**Response:**Thank you very much for this reminder. We removed the following sentence: “However, few studies have focused in discussing with the patient benefits and risk before the first prescription” and changed“Some authors consider that discussing the benefits and risk with the patient before the first prescription could be considered a key component in preventing long-term prescriptions of BZDs.”

Methods
The study is described as Type 1 trial, which implies a primary outcome that relates to clinical or health outcomes. The primary outcome is a medication prescription rate, which seems to me as aspect of physician behaviour, thus Type 3 hybrid effectiveness-implementation trial.

**Response:**Thank you for highlighting this important point, which has been an important point of discussion by the authors.

We previously conducted a randomized clinical trial that examined the effectiveness of an intervention on withdrawal from BZDs by long-term users (Vicens et al BJP 2014). The results indicated a significant reduction in BZD use by these individuals at 12 months. The aim of the current study was to evaluate the effectiveness of a more complex intervention that included adequate prescribing information, audit and feedback, and implementation of the intervention under more ‘real life’ conditions (i.e., all GPs from most PHCs`s participants health districts). At the same time we assessed fidelity to the intervention and the barriers and facilitators of implementing the intervention as perceived by the GPs.

This ‘type 1 hybrid design’ — testing the effects of a clinical intervention on relevant outcomes while observing and gathering information on implementation — seemed the best approach to achieve our objectives. Previous studies evaluated the effectiveness of an audit and feedback intervention on reducing GPs referrals for radiography (Eccles et al., 2001) and antibiotic prescriptions (Elouafkaoui et al, 2016). We focused primarily on the effectiveness of the intervention; the unit of randomization and measurements were at the provider level. However, we agree with the reviewer that some of the design elements and key challenges, as defined by Curran et al., could lead to classification of our study as a ‘type 3 hybrid design’.

There is still some debate among the authors regarding this issue. We could change the description to type 3, keep it as type 1, or simply describe the design as a cluster randomized clinical trial. We would be greatly pleased to hear your valuable opinion in this respect.

*Eccles M, Steen N, Grimshaw J, Thomas L, McNamee P, Soutter J, Wilsdon J, Matowe L, Needham G, Gilbert F, Bond S. Effect of audit and feedback, and reminder messages on primary-care radiology referrals: a randomised trial. Lancet. 2001 May 5;357(9266):1406-9.*

*Elouafkaoui P, Young L, Newlands R, Duncan EM, Elders A, Clarkson JE, Ramsay CR; Translation Research in a Dental Setting (TRiaDS) Research Methodology Group. An Audit and Feedback Intervention for Reducing Antibiotic Prescribing in General Dental Practice: The RAPiD Cluster Randomised Controlled Trial. PLoS Med. 2016 Aug 30;13(8):e1002115.*

The stratification component of the randomization is not quite clear, because it seems to relate to geographical districts rather than practices.

**Response:**Thank you for identifying this inconsistency. We made the following change to the randomization section:

“All PHCs were randomized at the same time to maintain allocation concealment and were stratified by health districts, PHCs baseline DDDs per 1000 inhabitants-per-day, and proportion of patients older than 65 years to ensure PHCs were balanced to those characteristics

I would recommend to describe the implementation strategies (education, feedback, info-platform) in more detail, using a reporting guideline such as TIDIER as appendix to the manuscript.

**Response:**Thank you. We included the TIDieR checklist and expanded this section based on the changes suggested by the academic editor (point #17 and especially point #22):

The multicomponent BENZORED intervention consisted of an educational meeting for GPs, audit and feedback, and a support web page.

Educational meetings are commonly used for continuing medical education with the aim of improving professional practice and patient outcomes. Educational meetings include courses, conferences, lectures, workshops, seminars, and symposia.[40]

At the start of the trial, the GPs from the intervention group received a 2-hour educational face to face workshop in their PHCs. These interventions were delivered by researchers who were GPs, had great expertise in prescribing and deprescribing BZD, and provided training about appropriate procedures for prescribing BZDs. This included educational information about the pharmacological properties of BZDs (biological half-life and equivalent dose of different BZDs), indications for prescription, recommended duration of use, adverse effects, dependence, tolerance, prevalence, and consequences of long-term use. These GPs were asked to discuss with their patients harms and benefits of taking BZD before the first prescription. They received training in a structured BZD discontinuation intervention based on gradual BZD dose reduction and training based on real clinical cases.[1,33]

After the initial training workshop, all participating GPs received automated monthly feedback. The audit and feedback, based on the “Feedback Intervention Theory”[45], provided a summary of the clinical performance of health care over a specified period of time that was aimed at changing the practices of health professional.[41]

Graphical depictions of BZD prescription feed-back consisted of a line graph that plotted each GP’s monthly BZD prescription rate in DDDs per 1000 inhabitants per day for 12 months, and two additional line graphs that plotted the BZD prescription rate of the PHC and the health district. The GPs also received a password to access to a support web page that provided additional information to reinforce the messages of the workshop. This web site included videos, descriptions of practical cases, and an information leaflet for patients about BZDs, Z-drugs, and sleep hygiene (http://benzored.es).

The number of outcomes in this manuscript is 3, which is rather. It seems that information on intervention fidelity is not available or not reported, but would be helpful to interpret the findings.

**Response:**Thank you. We expanded this section in the Results based on the changes suggested by the academic editor (point #22).

“Implementation process.

Full details of the methods used for evaluation of the implementation of the BENZORED IV intervention were reported elsewhere.[49] To summarize, 40 semi-structured interviews and 5 focus group meetings were conducted. GPs were invited to participate in an effort to ensure representativeness for the different health district locations, and they were classified as ‘low prescribers’ or ‘high prescribers’ based on the 12-month final evaluation.

The Consolidated Framework Implementation Research (CFIR) was used to guide development of the focus group meetings and for coding and data analysis. [42] The CFIR is a theoretical framework that provides a list of 41 constructs organized in five domains that can negatively or positively influence implementation.

Among the 45 GPs contacted, 40 agreed to interviews.

The implementation fidelity was measured as adherence to the intervention, defined as whether "a program service or intervention is being delivered as it was designed". [50] Adherence was measured using a questionnaire given to all GPS in the intervention group. This questionnaire asked the GPs to rank adherence to each of the following components of the intervention on a scale from 1 to 10: discussing benefits and risk with the patient before the first prescription; setting limits for the duration from the start of treatment; tailoring stepped dose reduction to discontinue BZD use in long-term users; reviewing BZD prescription feedback; downloading and using the patient information leaflet about BZDs, Z-drugs, and sleep hygiene; and visiting the web page to reinforce information.

Result section

Evaluating the implementation process

A total of 246/372 (66%) GPs from the intervention group answered this questionnaire. These GPs generally valued the multicomponent intervention as flexible and integrable into their practices. However, the three components of the intervention were valued differently. They reported the educational workshop was useful for clinical practice and the BZD prescription feedback was easy to interpret. They reported the tailored stepped dose reduction regimen for discontinuing BZDs was complex, and their lack of time and workload were significant barriers to implementation. The material included in the web page was considered helpful for the patients, but they reported some technical problems in accessing the website. The fidelity of adherence to the intervention differed among the different components. Discussing benefits and risk with the patient before the first prescription had a median adherence score of 8 (interquartile range [IQR]: 7 to 9); setting limits for the duration from the start of the treatment had a median score of 8 (IQR: 7 to 9); tailoring stepped dose reduction to discontinuing BZD use in long-term users had a median score of 7 (IQR: 7 to 8 ); reviewing BZD prescription feedback had a median score of 7 (IQR: 6 to 8); downloading and using the patient information leaflet about BZDs, Z-drugs, and sleep hygiene had a median score of 7 (IQR: 7 to 8); and visiting the web page to reinforce information had a median score of 5 (IQR: 3.75 to 7).

Results
A participation rate of 90% of invited practices is very high compared to many other studies in primary care. The authors may discuss this (in the discussion section).

**Response:** We appreciate the reviewer’s comment . We expanded the Discussion as suggested:

“To our knowledge, this is the largest clinical trial to analyze the effectiveness of an educational intervention for GPs in a primary care setting that focused on reducing prescriptions for BZDs. We included all GPs of the participating PHCs. The study had a high rate of GP participation compared to similar studies, and very few GPs were lost to follow up.”

While the study is large and seems well-conducted, the manuscript does not present many results

Discussion
Similar to the introduction section, the discussion could relate more to the implementation science literature, e.g. Cochrane review on audit and feedback and literature on de-implementation (stopping practices) in healthcare.

**Response:** As suggested, we compared our results to similar implementation strategies:

The overall reduction in BZD prescriptions that we observed in the intervention group was similar in magnitude to that found in an intervention that included education with audit and feedback.[40,41]

“Changing inappropriate health interventions is essential for minimizing patient harm, maximizing efficient use of resources, and improving population health. Prescribing BZDs in patients aged 65 years or more is considered inappropriate and should be avoided.”

Michel Wensing

**Reviewer #2:**

**Alex McConnachie**

**Statistical Review**

Vicens et al presents a report of BENZORED, a cluster randomised trial of an educational and feedback intervention for GPs to reduce benzodiazepine prescriptions. This review looks at the use of statistics in the paper.

Overall, I found this to be a nicely written paper. The statistical methods are generally good, with appropriate methods used to account for the cluster randomised design. I do have a few comments, which are generally minor, and should not affect the underlying message of the paper.

Much is made of the "Intention to Treat" analysis. This appears to have been interpreted as an analysis with multiple imputation to account for missing outcome data for some GPs. For me, this is not what ITT means. As the term implies, ITT is about analysing data from a randomised trial according to the intervention that was intended to be delivered. I.e., analysis according to the randomised allocation, regardless of whether the intervention was delivered as planned. In an RCT, all participants should be followed up, even those who withdraw from the intervention.

Loss to follow-up in randomised trials is common, but has nothing to do with the concept of ITT. In my opinion, the primary analysis should be limited to those GPs with follow-up data.

This can still be labelled as "ITT", so long as the GPs are analysed according to their original randomised allocation. The amount of missing follow-up data should be reported, and the reader can judge the validity of the primary analysis in the context of this missing data.

#### **Response:**Thank you for your kind review. We agree that intention-to-treat (ITT) analysis corresponds to analyzing the groups exactly as randomized. However, missing outcomes should also be addressed in the ITT analysis to "include all randomized participants in the analysis”, as described in the Consort statement: <http://www.consort-statement.org/checklists/view/32--consort-2010/96-statistical-methods>:

#### Intention-to-treat analysis

*The special strength of the RCT is the avoidance of bias when allocating interventions to trial participants (see box 1). That strength allows strong inferences about cause and effect that are not justified with other study designs. In order to preserve fully the huge benefit of randomization we should include all randomized participants in the analysis, all retained in the group to which they were allocated. Those two conditions define an “intention-to-treat” analysis, which is widely recommended as the preferred analysis strategy. Intention-to-treat analysis corresponds to analyzing the groups exactly as randomized. Strict intention-to-treat analysis is often hard to achieve for two main reasons—missing outcomes for some participants and non-adherence to the trial protocol.*

We agree with the reviewer that a complete case analysis and a sensitivity analysis is perfectly valid because only 6.5% of GPs were lost to follow up. However, our predefined protocol lists ITT analysis as the primary analysis based on the CONSORT recommendations.

As a sensitivity analysis, to assess the impact of missing data on the primary analysis result, multiple imputation is good method to use. However, I always feel that including the randomised group in the imputation process makes this circular; if the outcomes are imputed based on the intervention effect observed in those with complete data, this will tend to reinforce the complete case analysis. I would rather see the multiple imputation carried out without randomised group as a predictor, to see whether the primary analysis result is robust to the assumption of no intervention effect in those with missing outcomes.

**Response:**Thank you for this suggestion. We used multiple imputation without the randomized group as a predictor, and changed the ITT estimated parameters in the manuscript.

That is probably my main point, and is one of emphasis more than anything. Looking at the tables, the complete case analysis appears to give similar results to what is currently described as "ITT". Given the relatively good follow-up achieved in the study, I would not expect multiple imputation without use of randomised group as a predictor to eliminate the intervention effect.

My others points are more minor.

The background reports a figure of 87.6 DDDs per 1000 patients, and a figure of 8.76% of patients taking a DDD each day. These are two different things, so if this is true, then it is quite a coincidence. E.g. 87.6 DDDs per 100 patients could represent about 175 in every 1000 patients (17.5%) all consuming half a DDD each day, or 4.4% of patients taking 2 DDDs every day.

**Response:**We agree that this original text was not clear. To provide further clarification, we added the following text:

“The Spanish Medicine Agency (AEMPS) reported an average of 87.6 DDDs per 1000 inhabitants per day during 2018 (19). Thus, approximately 9% of the population of Spain is receiving an average daily equivalent of 10 mg of diazepam. According to the most recent Spanish health survey, an average of 10% of the population in Spain reported consuming BZD during the 2 weeks prior to the survey. Women aged 65 years or more were the highest consumers (36%) (20).”

I think the paper would benefit from a clearer statement about the unit of randomisation (PHC) and the unit of analysis (GP). It becomes clear as you go through the paper, but is not immediately obvious. For example, the sample size section talks about data aggregated at the cluster level (PHC), but then makes an assumption about the ICC, which implies analysis of data by GP, clustered within PHCs.

**Response:** Thanks for identifying this lack of clarity. We changed the sample size section to improve clarity:

“The sample size calculation was based on DDDs per 1000 inhabitants per day. The mean DDDs was 89.3, the standard deviation was 18.7, and the intra-cluster correlation coefficient was 0.05 (44).”

We also included a definition of the unit of randomization in the design section:

“Allocation was performed at the level of the primary healthcare centers (PHCs) to reduce bias due to ’treatment contamination”.”

The primary analyses used Poisson regression, but the intervention effects are reported as mean differences. How were the regression results converted to absolute differences?

**Response:**We calculated the average marginal effect of group (x1) on the predicted probability of the total BZD prescriptions. In particular, we used the Stata command margins: margins, dydx(x1) x1 = Arm

There is mention of the trial being reported according to the CONSORT guideline for cluster trials, but I did not see a checklist. One feature that is missing from the results is a report of the ICCs observed. This would be useful for future researchers.

**Response:**We included the CONSORT checklist for cluster randomized trials and reported ICC values as suggested by the academic editor (point #3)

“At the PHC level, the intra-cluster correlation coefficient of DDDs per 1000 inhabitants per day of BZDs was 0.30. This was higher than expected from the sample size calculation, indicating substantial within-PHC clustering for BZD prescriptions.”

I do not think it is important to report Cohen's d. The outcomes are easily interpretable as they are.

**Response:**Thanks for your comments. We removed mention of Cohen`s d.
