# Supplementary material for: Evaluation of a multicomponent intervention consisting of education and feedback to reduce benzodiazepine prescriptions by general practitioners: The BENZORED hybrid type 1 cluster randomized controlled trial
Source: PLoS Med. 2022 May 6;19(5):e1003983. doi: 10.1371/journal.pmed.1003983 (PMC9075619; doi:10.1371/journal.pmed.1003983)
Supplement: Attachment — Submitted filename: respuesta plos medicine final 14 feb.docx [file pmed.1003983.s009.docx]

Dear Caitlin Moyer (Associate Editor)

Thank you very much again for your helpful comments to our manuscript, “Effectiveness of a multicomponent intervention consisting of education and feedback on reducing benzodiazepine prescriptions by general practitioners: BENZORED hybrid type 1 cluster randomized controlled trial” (PMEDICINE-D-21-03642R2).

We provide our replies to the comments below and carefully revised the manuscript accordingly.

Thank you very much for your time and interest.

We look forward to hearing from you soon.

Sincerely,

Alfonso Leiva

Thank you for inviting us to submit a revised draft of our manuscript. We also appreciate the time and effort the academic editor and reviewers have dedicated to providing insightful feedback to strengthen our paper. We resubmit our article for further consideration. We have incorporated changes that reflect the detailed suggestions

I. **Requests from the editor:**
1. **Data availability statement:** **Thank you for providing the link to access the dataset. Please revise the statement to “All data are available from the Zenodo repository (https://ddec1-0-en-ctp.trendmicro.com:443/wis/clicktime/v1/query?url=https%3a%2f%2fdoi.org%2f10.5281%2fzenodo.5607352%29.%e2%80%9d&umid=52464d2c-2704-4151-86f1-45e4a1c05ca3&auth=9dc7eecf3108cde86575e4e15f505c977593cb03-fe4b407eb787668c7ed04ec86fddcc7158d14701 or similar. There may be a typo in the DOI provided, please check if this should be:** [https://ddec1-0-en-ctp.trendmicro.com:443/wis/clicktime/v1/query?url=https%3a%2f%2fdoi.org%2f10.5281%2fzenodo.5607352&umid=52464d2c-2704-4151-86f1-45e4a1c05ca3&auth=9dc7eecf3108cde86575e4e15f505c977593cb03-c90ed9cf2397a822854b3d1eadf6dc4272b8a8bc](https://ddec1-0-en-ctp.trendmicro.com/wis/clicktime/v1/query?url=https%3a%2f%2fdoi.org%2f10.5281%2fzenodo.5607352&umid=52464d2c-2704-4151-86f1-45e4a1c05ca3&auth=9dc7eecf3108cde86575e4e15f505c977593cb03-c90ed9cf2397a822854b3d1eadf6dc4272b8a8bc). **Please also check that no identifying information (GP, PHC name) are included in the file.**

**Response:** We uploaded a new version of the dataset with no identifying information. <https://doi.org/10.5281/zenodo.5970482>

2. **Abstract: Please structure your abstract using the PLOS Medicine headings (Background, Methods and Findings, Conclusions). Please incorporate the sentence describing the study objective as the final sentence Background.**

**Response:** As suggested we included the Background, Methods and Findings, Conclusions headings in the abstract

3. **Abstract: Methods and Findings: Please mention that analyses were intention to treat.
   Response:** As suggested we included that all analysis were by intention to treat principles.

We included in the abstract: “Intention to treat (ITT) analysis was used to assess all clinical outcomes”

**Abstract: Methods and Findings: Line 121-122: Please mention the characteristics for which the two groups were not apparently balanced.**

**Response:** As suggested we included the main limitations of the study and also, we mention the characteristic for which the two groups were not balanced in the abstract, please note we move this paragraph from the first paragraph to the last sentence of the Abstract methods and findings.

“A key limitation of this clustered design clinical trial is the imbalance of some baseline characteristics. The control groups have a higher rate of baseline BZD prescription, and more GPs in the intervention group were women, physician doctors, and trainers of GP residents”

4. **Abstract: Methods and Findings: In the last sentence of the Abstract Methods and Findings section, please describe the main limitation(s) of the study's methodology.**

**Response:** as mention above we included in the last sentence of the abstract:

“A key limitation of this clustered design clinical trial is the imbalance of some baseline characteristics. The control groups have a higher rate of baseline BZD prescription, and more GPs in the intervention group were women, physician doctors, and trainers of GP residents”

5. **Throughout: Please place the in-text citations within square brackets before the sentence punctuation, for example [1].**

**Response:** As suggested we placed the in-text citations within square brackets before the sentence punctuation

6. **Throughout: Please carefully check the text for typos/grammatical errors.**

**Response:** As suggested we reviewed the manuscript checking for possible grammatical errors.

7. **Methods: Line 239: Please include a copy of the original study protocol as a supporting information file, and please refer to it here (for example, S1 Protocol).**

**Response:** As suggested we included the reference to the study protocol as a supporting information

“The details of the protocol were published elsewhere[44] (S1 Study Protocol).”

8. **Methods: Line 244: Please include the information regarding participant (PHC) consent and include the information that the ethical review boards waived the requirement for GP consent.**

**Response:** My apologies, as the corresponding author I did not personally check the three ethical approvals, after verifying them, the Balearic Islands and the IDIAP Jordi Gol Ethics Committee waived the informed consent. However, the Valencian Primary Care Ethics Committee did not, therefore we only included PHCs in the Valencian area if the GPs signed the informed consent**.**

We have included as suggested: “Only PHCs in which at least two-thirds of the GPs agreed to participate were included. Informed consent was waived by the Balearic Islands and the l'IDIAP Jordi Gol Ethical Committee of Clinical Research because the analysis used anonymous administrative data.”

9. **Methods: Line 342: Please reference the analysis plan in the Supporting Information files here.**

**Response:** As suggested we included the reference to the analytic plan:

“All statistical analysis were performed using SPSS v.23.0 (IBM Corp., Armonk, NY, USA) using a predefined analysis plan (S1 Analytic plan)..”

10. **Methods: Line 344-345: Please revise the CONSORT statement to: "These results are reported as per the Consolidated Standards of Reporting Trials (CONSORT) guideline extension for cluster randomized trials (S1 CONSORT Checklist)."**

**Response:** As suggested we included (S1 CONSORT Checklist)

11. **Methods: Line 362: Please provide a copy of the questionnaire as a supporting information file.**

**Response:** As suggested we included the fidelity questionnaire as supporting information file.

“Adherence was measured using a questionnaire given to all GPS in the intervention group (S1 Implementation Fidelity Questionnaire).”

12. **Results: Line 401: Please summarize the key findings from the additional analyses of effects by health district.**

**Response:** As suggested we included the reported effect sizes by health district:

“The adjusted differences in the total DDDs per 1000 inhabitants-per-day between groups in the Tarragona-Reus was -6.8 (95%CI -12.2 to -1.30, p=0.023), in the Arnau de Vilanova llíria was -3.3 (95%CI -7.5 to 1.2, p=0.156) and in the Balearic islands (ib-salut) was -2.8 (95%CI -4.3 to -1.3, p <0.001)”

13. **Discussion: Line 455-456: Please provide references and clarify this statement: “Prescribing BZDs in patients aged 65 years or more is considered inappropriate and should be avoided.”**

**Response:** Thank you for your comments, we have clarified and referenced the statement as follows:

“Prescribing BZDs in patients aged 65 years or more is considered potentially inappropriate and should be avoided because the decreased metabolism of long-acting agents and increased sensitivity and risk of cognitive impairment, delirium, falls, fractures, and motor vehicle crashes in older adults[14].

14. **Checklists: Thank you for including the 3 reporting checklists. Please revise the checklists, using section and paragraph numbers to refer to locations within the text (e.g. Methods, paragraph 1). Please do not refer to page numbers.**

**Response:** As suggested we include section and paragraph numbers

**16. Supporting information Table 1: Please provide a legend for this table.**

**Response:** As suggested we include a legend for Table 1.

“Table1. Subgroup analysis of effectiveness of the intervention at 12 months follow-up by health districts”

**17. Analysis Plan: Please note at what point during the study the amendment to the analysis plan was made.**

**Response:** As suggested we included it in the supporting information the date of the Final Statistical Analysis Plan and amendments

“Date of Final Statistical Analysis Plan: 07/01/2016 Amendment No. 1: 23/08/202”

II. **Comments from the reviewers:

    1. Reviewer #2: Alex McConnachie, Statistical Review

    I thank Vicens and colleagues for their consideration of my original points.

    On the matter of the meaning of ITT, I think we can agree to disagree. I accept that I may be in the minority in my opinion, even if I think I am right!

    However, this got me thinking about the analysis. By imputing the missing outcomes for GPs who moved out of the PHC area, the analysis is estimating the effect of the intervention under the scenario that all GPs remained within their original PHC. I am not sure this is realistic.**

**Response:**  **We agree with the reviewer that there is not an ideal option here; there is, in effect, a choice between omitting participants without final outcome data or imputing the missing outcome data.**

**Douglas G Altman also address this dilemma “ What should researchers do? A “complete case” (or “available case”) analysis simply omits those for whom data are incomplete. This commonly used approach loses power, and bias may well be introduced, given that the incompleteness of data will not be random. Further, excluding some patients is not compatible with the ITT principle……..”(*Altman D. Missing outcomes in randomized trials: addressing the dilemma. Open Medicine. 2009;3(2):e51.].***

**Also, in the analysis, it appears that only those who expressed an interest in the study prior to randomisation are included. Therefore, the analysis is estimating the impact of the intervention in a subgroup of GPs, rather than the overall impact in the population of all GPs. If implemented, some GPs might decline the intervention, and these GPs are not being included in the analysis.**

**Since randomisation took place at the PHC level, the fairest analysis, under ITT, might be to include all GPs within each PHC, regardless of whether they received the intervention, since the randomisation resulted in the intervention being available in some PHCs but not others.**

**Since outcomes were collected from electronic records, would it have been possible to collect complete outcome data for all GPs in each PHC at baseline and at follow-up, removing the need for imputation of missing data?**

**I suppose one problem with this approach would be that for those GPs who move into an area during the study, there would be no baseline value for the outcome variables, making the analysis at GP level more difficult.**

**Response: We thank the reviewer for the insightful comments. It was not possible to collect the follow up data of those GPs that moved out of the PHC area. A GP that moved to other PHC is assigned new patients then baseline data and follow up date are from completely different sets of patients.**

**All this being said, I think that what the authors have done is very good. Both the complete case and multiple imputation results are presented and are similar. Given that GPs who were unwilling to take part were excluded prior to randomisation, what are the implications in terms of the population-level effect of the intervention? Did these GPs, in intervention PHCs, have access to the intervention? If so, did they receive it, or did they tend to decline?**

**Response: Thanks for your comments. GPs were not excluded prior to randomization. All GPs from PHC that accept to participate were included. However not all PHC accepted participation, even the high rate of participation (90%) PHC that declined to participate could be different that those that accepted participation.**

**.**

**The method of estimating the marginal mean difference in DDDs from a Poisson model is fine, but should be mentioned in the statistical methods section.**

**Reponse:** **We agree and we included as the reviewer suggest**:

**“For the primary outcome the effectiveness of the intervention was analyzed using a mixed effects Poisson regression model to account for clustering at the level of the PHC and adjusted for baseline GPs DDDs per 1000 inhabitants-per-day. Also, adjusted average marginal effects for group were calculated from the mixed effects Poisson regression models”**

**Finally, I still have a problem with the text "The Spanish Medicine Agency (AEMPS) reported an average of 87.6 DDDs per 1000 inhabitants-per-day during 2018. Thus, approximately 9% of the population of Spain is receiving on average daily equivalent of 10mg of diazepam." I do not believe that it is possible to deduce the percentage of the population who are receiving a medication, based on the average DDDs per 1000 participants. Approximately 90 DDDs per 1000 population could represent 9% of the population receiving an average of 1 DDD each, or it could represent 18% of the population receiving an average of 0.5 DDDs each. There is no way to tell.**

**Response: Thanks for your comments. We removed the sentence form the background section**

**2. Reviewer #3: Thank you for revising the manuscript. I don't have any further points to raise.**


Any attachments provided with reviews can be seen via the following link:

__________________________________________________
In compliance with data protection regulations, you may request that we remove your personal registration details at any time.  (Use the following URL: [https://ddec1-0-en-ctp.trendmicro.com:443/wis/clicktime/v1/query?url=https%3a%2f%2fwww.editorialmanager.com%2fpmedicine%2flogin.asp%3fa%3dr&umid=52464d2c-2704-4151-86f1-45e4a1c05ca3&auth=9dc7eecf3108cde86575e4e15f505c977593cb03-272226d1b5035a49ef6175b42cf8bcae59323664)](https://ddec1-0-en-ctp.trendmicro.com/wis/clicktime/v1/query?url=https%3a%2f%2fwww.editorialmanager.com%2fpmedicine%2flogin.asp%3fa%3dr&umid=52464d2c-2704-4151-86f1-45e4a1c05ca3&auth=9dc7eecf3108cde86575e4e15f505c977593cb03-272226d1b5035a49ef6175b42cf8bcae59323664)). Please contact the publication office if you have any questions.
