# Supplementary material for: Evaluation of a multicomponent intervention consisting of education and feedback to reduce benzodiazepine prescriptions by general practitioners: The BENZORED hybrid type 1 cluster randomized controlled trial
Source: PLoS Med. 2022 May 6;19(5):e1003983. doi: 10.1371/journal.pmed.1003983 (PMC9075619; doi:10.1371/journal.pmed.1003983)
Supplement: Attachment — Submitted filename: Carta para Plos ultima revision 22 Marzo sin comentarios.docx [file pmed.1003983.s010.docx]

From the academic editor:

-Abstract: Methods and Findings Line 125: As the primary outcome is reported as absolute difference, please similarly report the secondary outcome as absolute rather than relative difference: “The intervention group also had a smaller number of adjusted relative long-term users overall (-3,6% (95%CI: 1.85,8.88, p>0,001) and a smaller number of adjusted relative long-term users over age 65 years (.- 3.5% (95%CI: 1.19, 5.71 p=0,004)”

**Response:**As suggested, we have included absolute differences.

“The intervention group also had a smaller number of long-term users. The adjusted absolute difference overall was -0.36 (95%CI: -0.55,-0.16, p>0,001) and the adjusted absolute difference in long-term users over age 65 years was -0.87 (95%CI: -1.44,-0.30, p=0,003).”

-Methods: Study population: Line 248: I think it might be helpful to insert a brief few sentences on how PHC is organised in Spain (regions, districts, PHCs, GPs).

**Response:** Thanks, we added this information in study population.

“Healthcare in Spain is a public service with universal coverage and free access for the entire population. Spanish primary care system consists of three organizational levels: The State´s central administration agency that is responsible of the general coordination and legislation and the pharmaceutical policy; the delivery of health services are responsible of regions (Autonomous communities) and regions are organized by health districts that includes PHCs. PHCs are staffed by multidisciplinary teams comprising of GPs, pediatricians, nurses, gynecologists, and physiotherapists.

-Methods: Line 305: The second secondary outcome measure is perhaps better considered a sub-group analysis (people over 65 years who are long term BZD users). Was there any heterogeneity of effect between those >65 years and those <=65 years? It looks like there was also a pre-specified sub-group analysis by district (e.g. described at line 339-341 and reported at line 406). It would be good to have a sub-heading on pre-specified sub-group analyses and ensure all are reported in the results.

**Response:** Thanks for the comments. The proposed subgroups analysis of the study include Gp characteristic as sex and years working, characteristic of the patient listed ( percentage of patient aged 65 years old and sex) and health district. We have included in the manuscript as a subheading. We carried out a formal subgroup analysis introducing the interaction term between the total BZD prescription and these characteristic. The subgroups analysis should be limited to the primary outcome (total BZD prescription) and the proposed subgroup analysis (>65 years vs <=65 years long-term users) involved a secondary outcome.

“Subgroup analysis: To assess whether the effect of the intervention on BZD prescription varied according to GP characteristics (sex and working years), characteristic of the GP`s practice list (percentage of patient aged 65 years old and sex) and health districts, a subgroup analysis of effectiveness that was previously planned in the protocol was performed. “

-Results, Table 2: Please include a footnote to describe what covariates were included in the adjusted model. It is difficult to work out from the main manuscript text.

**Response:** We thank the academic editor for highlighting this point. We included in the legend as suggested in point 33.

“Comparison of General Practitioners’ benzodiazepine prescriptions, percentage of all BZD long-term users (≥ 6 months) and percentage of BZD long-term users aged 65 or more. Unadjusted, adjusted (DDD per 1000 inhabitants per day and percentage of BZD long-term users at baseline) and estimated ITT results for the control and intervention groups at 12-month follow-up.”.

-Results: I am a little surprised that with the large actual ICC (0.3) relative to the sample size calculations (0.05) that the study was adequately powered to show a difference – the effect size is smaller and the number of general practices is only a little higher compared to the original calculations. But it is hard to calculate the actual study power based on the data providers (e.g the patient cluster sizes at the PHC level are not given). Could this be checked?

**Response:** In our study GPs (not patients) are clustered at the PHC level GPs, the average cluster size was 10 GPs (please note the patient cluster size in this case is irrelevant because our analysis include aggregate patient data). We were also surprised by the large ICC of GP`s prescription within PHCs. As the academic editor point out, it could affect the study power (type II error) “the probability of correctly rejecting the null hypothesis when it is false” (likelihood of a significance test detecting an effective intervention when the intervention actually is effective). However, the type II error has only relevance when null hypothesis is not rejected and in our study we already rejected the null hypothesis.

-Results: Also related to the point above it would be helpful to understand a little more what is happening at the PHC level to cause such a high level of clustering? This is related to my comment above on PHC structure in Spain. Most of the discussion focuses on the level of GPs and while I can understand within general practice clustering I am a little unclear what is driving PHC level clustering.

**Response:** As we mention before. In our study GPs are clustered within PHC. There is little heterogeneity about GP’s BZD prescription within PHCs, this seemingly means that higher and lower BZD prescribers are clustered at PHC level.

Other editorial points:

1.Title: Please revise to: “Evaluation of a multicomponent intervention consisting of education and feedback to reduce benzodiazepine prescriptions by general practitioners: The BENZORED hybrid type I cluster randomized controlled trial” and please update this in the manuscript submission system as well as the text of the manuscript.

**Response**: As suggested, we changed the Title in the manuscript and in the submission system.

2. Data availability statement: We suggest revising to: “The de-identified data underlying the study’s findings are publicly available in the Zenodo repository: [https://ddec1-0-en-ctp.trendmicro.com:443/wis/clicktime/v1/query?url=https%3a%2f%2fdoi.org%2f10.5281%2fzenodo.5970482%e2%80%9d&umid=b7296519-3edd-4833-9f5b-883052a49f72&auth=9dc7eecf3108cde86575e4e15f505c977593cb03-e978c7e7228abd40c4a591915ea52b800e7d3b0e](https://ddec1-0-en-ctp.trendmicro.com/wis/clicktime/v1/query?url=https%3a%2f%2fdoi.org%2f10.5281%2fzenodo.5970482%e2%80%9d&umid=b7296519-3edd-4833-9f5b-883052a49f72&auth=9dc7eecf3108cde86575e4e15f505c977593cb03-e978c7e7228abd40c4a591915ea52b800e7d3b0e)

**Response:** As suggested, we changed the Data availability statement in the submission system

3. Throughout: Please edit for minor typos and grammatical errors throughout.

**Response:** Thanks for the suggestion, we have edited minor typos and grammatical errors.

4. Abstract: Line 112-113: “GPs were not blinded to the allocation.” Please also mention who was blinded to the allocation.

**Response:** We have included in the abstract.

“the pharmacists, researchers, and trial statisticians were blinded to the allocation arm”

5. Abstract: Line 114: Please clarify if “tapering” rather than “withdrawal” would be preferable here.

**Response:** As suggested, we changed the sentence

**“**tapering-off long-term BZD use using a tailored stepped dose reduction”

6. Abstract: Line 115: We suggest revising to: “...with monthly BZD prescription feedback and access to a support webpage.”

**Response:** As suggested, we changed the sentence.

7. Abstract: Line 116-117: Please clarify the number of GPs (and PHCs) lost to follow up in each group.

**Response:** As suggested, we have included in the abstract

“Fourty nine GPs (21 intervention group and 28 control group) were lost to follow up.”

8. Abstract: Line 119: Please define “long term users” in this sentence

**Response:** As suggested, we have defined long-term use.

“The two secondary outcomes were the proportion of long-term users (≥ 6 months)……)

9. Abstract: Line 131: Please provide a definition of “physician doctor” as this may not be a universally understood term.

**Response:** As suggested, we changed physician doctor by GP with a doctorate degree

“The control groups have a higher rate of baseline BZD prescription, and more GPs in the intervention group were women, GPs with a doctorate degree, and trainers of GP residents.”

10. Author Summary: Line 165-167: We suggest revising to: “... successfully reduced the proportion of long-term users, which could potentially reduce adverse outcomes related to long-term use.” or similar.

**Response:** As suggested, we changed the sentence.

“successfully reduced the proportion of long-term users, which could potentially reduce adverse outcomes related to long-term use.”

11. Line 172: Please change “Background” to “Introduction” as the heading for this section.

**Response:** As suggested, we changed “Background” to “Introduction.

12. In-text citations: Throughout, please include a space between the reference bracket and the preceding word, for example [1]. Please also check that all reference brackets are placed before the sentence punctuation, where applicable.

**Response:** As suggested, we checked and changed reference brackets.

13. Methods: Please clarify the nature of the informed consent provided by GPs at PHCs included in the study.“Only PHCs in which at least two-thirds of the GPs agreed and provided written informed consent to participate were included. Informed consent was waived by the Balearic Islands and the l'IDIAP Jordi Gol Ethical Committee of Clinical Research because the analysis used anonymous administrative data. The requirement for informed consent was not waived by the Valencian Primary Care Ethics Committee, and therefore, from this region, only PHCs from which all GPs agreed and provided written informed consent were included.” or similar, if this is accurate.

**Response:** As suggested we included in the methods section.

14. Methods: Line 294-295: “This web site included videos, descriptions of practical cases, and an information leaflet for patients about BZDs, Z-drugs, and sleep hygiene (https://ddec1-0-en-ctp.trendmicro.com:443/wis/clicktime/v1/query?url=http%3a%2f%2fbenzored.es%29.%e2%80%9d&umid=b7296519-3edd-4833-9f5b-883052a49f72&auth=9dc7eecf3108cde86575e4e15f505c977593cb03-e0df879296a3b5784c314e75eb629cd9269a77c7 Please note that the link provided does not seem to be functional, please include an accessible link

**Response:** We thank the editor for highlighting this point. The website is now accessible.

15. Methods: Line 300-301: “Total number of dispensed DDD from the N05BA, N05CD, and N05CF groups of the Anatomical Therapeutic Chemical (ATC) Classification System code…” please clarify what prescription drugs are included in these codes.

**Response:** We have included all the drugs of the therapeutic groups.

“Total number of dispensed DDD from the N05BA (diazepam, chlordiazepoxide, potassium clorazepate, lorazepam, bromazepam, clobazam, ketazolam, alprazolam, halazepam, pinazepam, clotiazepam, bentazepam), N05CD (flurazepam, flunitrazepam, triazolam, lormetazepam, midazolam, brotizolam, quazepam, lorprazolam) and N05CF (zopiclone, zolpidem, zaleplon) groups of the Anatomical Therapeutic Chemical (ATC) Classification System code.

16. Results: Line 378-380: We suggest clarifying to: “This study was conducted in three Spanish health districts, which included 90 PHCs (58 in the Balearic Islands, 20 in the Tarragona-Reus district, and 12 in the Arnau de Vilanova-Llíria district) and 867 eligible GPs (Figure 1).”

**Response:** As suggested we included in the result section.

17. Results: Line 391-392: Please report the baseline DDD per 1000 inhabitants per day for the intervention and control groups in the text.

**Response:** As suggested we included in the result section the baseline DHD of each group.

“However, the average DDDs per 1000 inhabitants-per-day was slightly higher in the usual care group, 74.6 in the intervention group and 76.9 in the control group, and more GPs in the intervention group were women…..”

18. Results: Line 418-425: Please mention in the text what factors were adjusted for in these analyses.

**Response:** As suggested we included in the result section.

“The between groups differences adjusted by baseline BZD prescription in the total DDDs per 1000 inhabitants-per-day in the Tarragona-Reus…..”

“..In particular, the adjusted by baseline BZD prescription, difference of long-term BZD users among”

19. Results: Line 417: For secondary outcomes, it would be helpful to report the absolute differences, as mentioned by the Academic Editor (please see the comment on the Abstract).

**Response:** As mentioned in the point number 1 we changed in the abstract as Academic Editor suggested.

20. Results: Line 427-436: Please present data on these results, e.g. percentage of respondents who reported on each item of the questionnaire. Please consider adding a table with complete results for these outcomes.

**Response:** The global response rate was 66% (as stated in the manuscript). It was a short questionnaire of 6 items and all questions were answered by responders.

21. Discussion: Line 459-462: “In contrast, other authors found that educational visits with GPs regarding BZD prescriptions were not effective in reducing the number of prescriptions[52,53]. Pimlott et al. and Holm also found that an educational intervention and feedback to GPs was not effective[54,55].” Some additional discussion on why these educational/feedback interventions may have been less effective would be helpful to provide context.

**Response:** The intervention in both studies were similar to our intervention, however there are some flaws in the design of both studies: insufficient sample size, lack of allocation concealment and unequal allocation. Both studies were clearly underpowered to detect differences and they were poorly randomized.

We have included in the manuscript.

“Pimlott et al. and Holm also found that an educational intervention and feedback to GPs was not effective [54,55]. However these studies have some methodological limitations.”

22. Discussion: Line 468-470: Please revise if it might be more accurate to interpret this as: “We found that our intervention led to a 3.5% reduction in the proportion of long-term BZDs users who were aged 65 years or more.”

**Response:** Thanks for the suggestion, we have changed the sentence.

23. Discussion: Line 472-473: We suggest revising to “A previous study of a stepped-dose reduction of BZDs, with written instructions delivered by GPs to long-term users…” or similar

**Response:** Thanks for the suggestion, we have changed the sentence.

24. Discussion: Line 477-479: Please clarify here and throughout, as the secondary analysis revealed a reduction in proportion of long-term BZD users, rather than a reduction in BZD use by long term BZD users. We suggest changing to: “These results differ from our finding that the intervention implemented by GPs provided only a modest reduction in the percentage of long-term BZD users.”

**Response:** Thanks for the suggestion, we have changed the sentence.

25. Discussion: Line 503-505: Please include slightly more context here, briefly discussing how these local policy differences could have influenced your results. Please also include relevant citations: “For example, the Balearic Islands and Tarragona have local policies regarding BZD prescribing that include indicators and incentives.”

**Response:** Local policies included indicators and incentives. However they are included in the health programs for GPs and they are only available for institutional purposes but not published anywhere. We have added

“indicators and incentives to motivate GPs to reduce their BZD prescription”.

26. Discussion: Line 506-507: We suggest revising to: “This suggests the results may be applicable in different clinical settings.”

**Response:** Thank you for this comment, we have changed the sentence.

27. Line 538: Please remove the “Author contributions” section from the main manuscript text, and ensure all information is completely entered into the relevant sections of the manuscript submission system

**Response:** As suggested we removed this section from the main manuscript.

28. Line 553: Please remove the “Funding statement” section from the main manuscript text, and ensure all information is completely entered into the relevant sections of the manuscript submission system.

**Response:** As suggested we removed this section from the main manuscript.

29. Line 564: Please remove the “Competing interests statement” section from the main manuscript text, and ensure all information is completely entered into the relevant sections of the manuscript submission system.

**Response:** As suggested we removed this section from the main manuscript.

30. Line 566: Please remove the Ethics and Dissemination section from the end of the manuscript, and ensure the information is included in the appropriate location of the Methods section (this information could be added to the “Study Design” sub-section, or as a separate sub-section).

**Response:** As suggested we removed this section from the main manuscript and check that all information was included in the study design subsection.

The sentence “All data are available upon reasonable request.” can be removed as the information on accessing the publicly available data is included in the Data Availability statement of the manuscript submission system.

**Response:** As suggested we removed this section from the main manuscript.

31. References: Please check the formatting of each reference in the list. Please use the "Vancouver" style for reference formatting, and see our website for other reference guidelines [https://ddec1-0-en-ctp.trendmicro.com:443/wis/clicktime/v1/query?url=https%3a%2f%2fjournals.plos.org%2fplosmedicine%2fs%2fsubmission%2dguidelines%23loc%2dreferences&umid=b7296519-3edd-4833-9f5b-883052a49f72&auth=9dc7eecf3108cde86575e4e15f505c977593cb03-68c06675d2ae1c052e6d2a5652578ca64bb285e8](https://ddec1-0-en-ctp.trendmicro.com/wis/clicktime/v1/query?url=https%3a%2f%2fjournals.plos.org%2fplosmedicine%2fs%2fsubmission%2dguidelines%23loc%2dreferences&umid=b7296519-3edd-4833-9f5b-883052a49f72&auth=9dc7eecf3108cde86575e4e15f505c977593cb03-68c06675d2ae1c052e6d2a5652578ca64bb285e8)

For example:

Reference 4: The journal title should be “BMJ”

Reference 6: The journal title is missing.

Reference 9: This reference is incomplete.

Reference 27: This reference is incomplete.

Reference 30: Please check the journal title.

**Response:** As suggested we have checked all references.

32. Table 1: Please define “GP Specialty Training” as this was not mentioned in the Methods (please describe assessment of GP characteristics in the Methods).

**Response:** As suggested we included assesment of PHC and GP characteristic in the method section.

“At baseline we assessed PHC characteristics (total number of patient listed, proportion of patients aged 65 years or more, urban/rural setting and training practice) and GP characteristics (age, sex, GPs with 3 years specialty training, doctorate degree, resident trainer, and years working as GP).”

We also defined in table 1 GP Specialty Training as “GP with 3 years specialty training”

33. Table 2: In the legend, it would be helpful to define that the table reports on the percentage of all patients who are long term BZD users (and how this is defined), and the percentage of long-term BZD users who are older than 65 years. Please mention the factors adjusted for in the analyses in the legend (i.e. please briefly describe the unadjusted, adjusted, and estimated ITT analyses).

**Response:** As suggested we added in tables 2 legend.

“Comparison of General Practitioners’ benzodiazepine prescriptions, percentage of all BZD long-term users (≥ 6 months) and percentage of BZD long-term users aged 65 or more. Unadjusted, adjusted (DDD per 1000 inhabitants per day and percentage of BZD long-term users at baseline) and estimated ITT results for the control and intervention groups at 12-month follow-up.”

34. Supporting Information Table 1: Please remove the duplicate file (the file without p values).

**Response:** As suggested we remove duplicate files.

35. TIDieR, StaRI, and CONSORT Checklists: Please remove the older versions of these files.

**Response:** As suggested we remove older versions.

36. S1 Implementation Fidelity Questionnaire: Please include a copy of the questionnaire as a supporting information file.

**Response:** As suggested we included a copy of the questionnaire as a supporting information file

37. Study protocol: Thank you for including the original copy of the study protocol (“renamed_11888”). Please include a version in English, and we suggest renaming the file (e.g. S1 Protocol, or similar).

**Response:** As suggested we included an English version of the protocol.

Comments from Reviewers:

Reviewer #2: Alex McConnachie, Statistical review

I thank the authors once again for their responses. The section about DDDs and percentage of users has been removed, which was my only real objection. Whilst I feel there are other ways that the analysis could have been done, I am happy with the analyses as presented, and I have no further comments to make.

Any attachments provided with reviews can be seen via the following link:

_____________
